# Supplementary material for: TFAP2C-Activated MALAT1 Modulates the Chemoresistance of Docetaxel-Resistant Lung Adenocarcinoma Cells
Source: Mol Ther Nucleic Acids. 2019 Jan 18;14:567–82. doi: 10.1016/j.omtn.2019.01.005 (PMC6374643; doi:10.1016/j.omtn.2019.01.005)
Supplement: Document S1. Figures S1–S5 [file mmc1.pdf]

OMTN, Volume *14*

Supplemental Information

TFAP2C-Activated MALAT1 Modulates the Chemoresistance of Docetaxel-Resistant Lung Adenocarcinoma Cells

Jing Chen, Xiaobei Liu, Yichen Xu, Kai Zhang, Jiayuan Huang, Banzhou Pan, Dongqin Chen, Shiyun Cui, Haizhu Song, Rui Wang, Xiaoyuan Chu, Xiaoli Zhu, and Longbang Chen

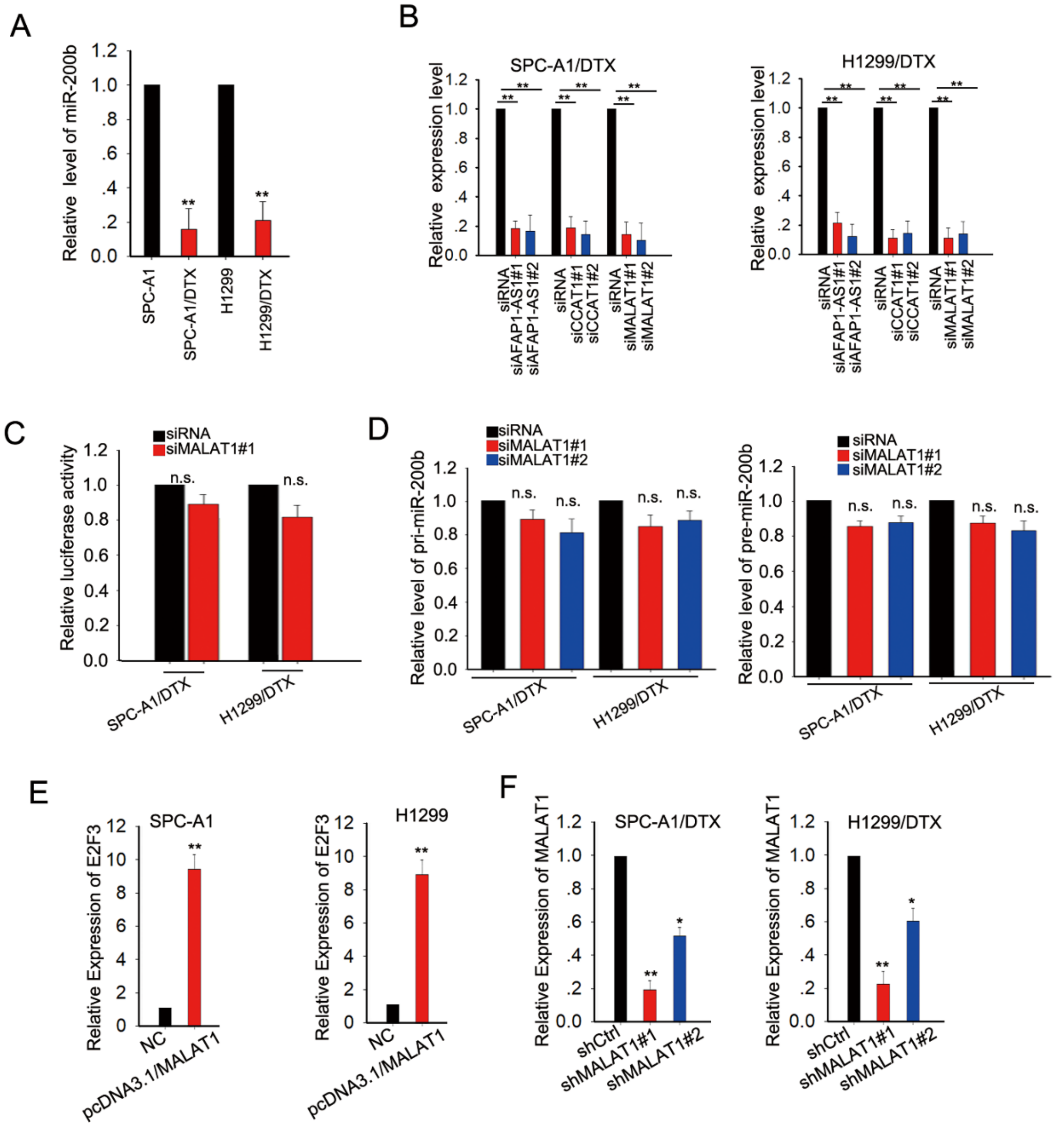

Supplementary Figure S1. The effects of

A. The miR-200b expression level in SPC-A1/DTX (H1299/DTX) and parental SPC-A1 (H1299) cells was determined by qRT-PCR. B. The knockdown efficiency of siRNAs specific to AFAP1-AS1, CCAT1 or MALAT1 was determined by qRT-PCR. C. The effect of MALAT1 knockdown on the miR-200b gene promoter activity was measured by luciferase reporter activity. D. The effect of MALAT1 knockdown on the expression levels pri-miR-200b and pre-miR-200b was measured by qRT-PCR. E. The transfection efficiency of pcDNA3.1/MALAT1 in parental cells and MALAT1-specific

shRNAs in DTX-resistant LUAD cells. **P < 0.01; n.s: no significance.

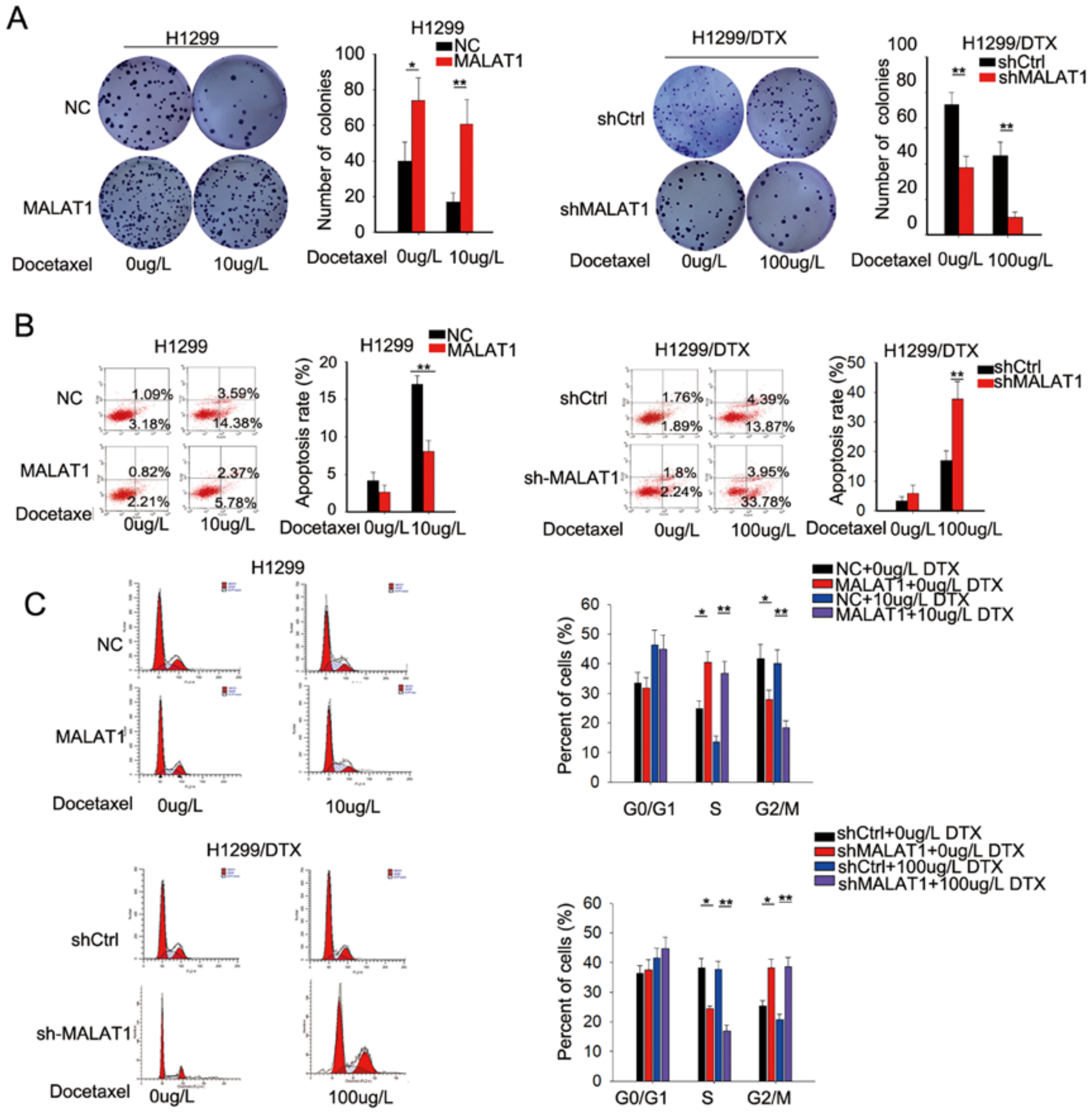

Supplementary Figure S2.

A. Representative colony formation results in H1299 cells transfected with MALAT1 expression vector and H1299/DTX cells transfected with MALAT1 shRNA. B-C. Flow cytometric analysis of apoptosis and cell cycle in H1299 cells transfected with pcDNA3.1 or pcDNA3.1/MALAT1 with or without treatment of DTX; or in H1299/DTX cells transfected with shRNA or shMALAT1 with or without treatment of DTX. *P < 0.05, **P < 0.01.

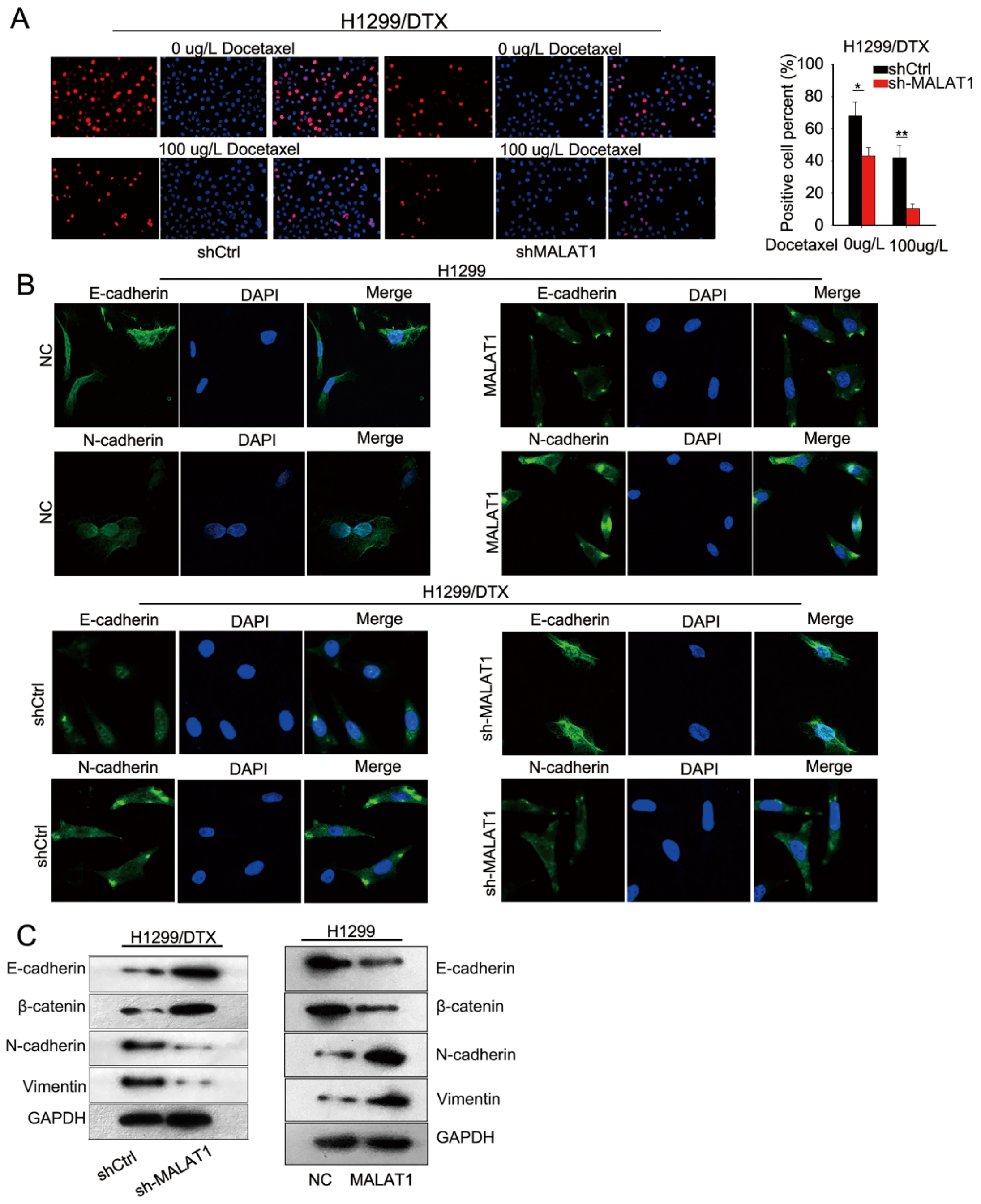

Supplementary Figure S3.

A. EdU proliferation assay in MALAT1-overexpressed H1299 cells or MALAT1-downregulated H1299/DTX cells. B. Immunofluorescence E-cadherin and N-cadherin staining was conducted in shRNA- or shMALAT1-transfected SPC-A1/DTX cells and pcDNA3.1- or pcDNA3.1/MALAT1-transfected SPC-A1 cells. C. E-cadherin and N-cadherin expression in shRNA- or shMALAT1-transfected H1299/DTX cells and

pcDNA3.1- or pcDNA3.1/MALAT1-transfected H1299 cells was determined by western blotting. *P < 0.05, **P < 0.01.

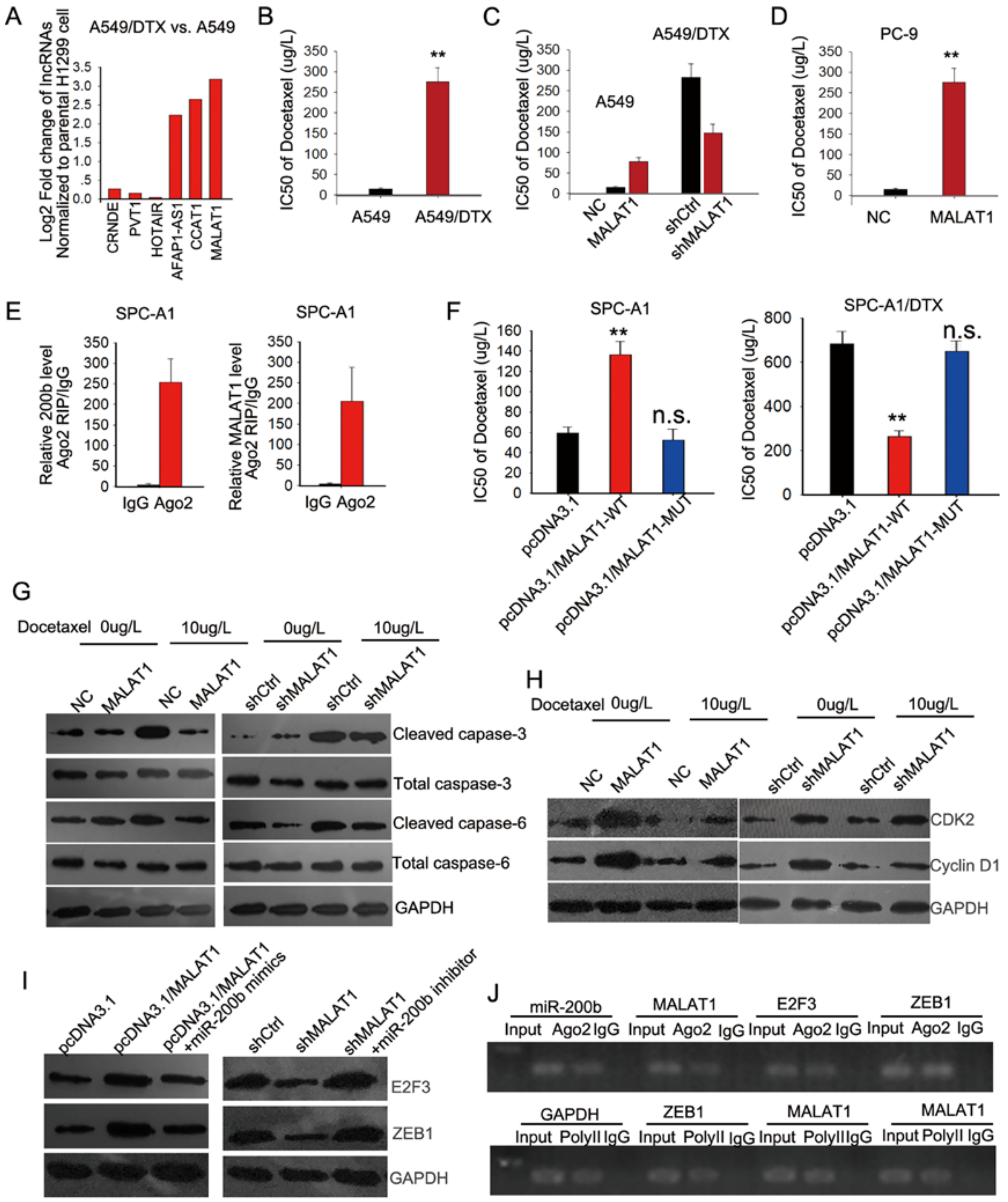

Supplementary Figure S4.

A. The fold change of six lncRNAs in A549/DTX and A549 cells. B. The IC_{50} of A549 and A549/DTX cells was tested with MTT assay. C. The IC_{50} was determined in A549

cell transfected with MALAT1 expression vector and NC or A549/DTX cell transfected with shMALAT1 and shCtrl. D. The IC_{50} value of PC-9 cell transfected with MALAT1 expression vector or NC. E. The interaction between MALAT1 and miR-200b in SPC-A1 cell was detected by RIP assay. F. The IC_{50} was examined in SPC-A1 cell transfected with pcDNA/MALAT1-WT, pcDNA/MALAT1-MUT or pcDNA-NC as well as in SPC-A1 cell transfected with shMALAT1-WT, shMALAT1-MUT or shCtrl. G-H. The expression levels of proteins related with cell cycle progress and apoptosis were tested by western blot analysis. I. The effects of MALAT1 and miR-200b on the protein levels of E2F3 and ZEB1 were determined by western blotting. J. Up: the gelling results for the qRT-PCR products of miRNA and mRNA; bottom: the DNA fragments for ChIP assay. **$P < 0.01$; n.s: no significance.

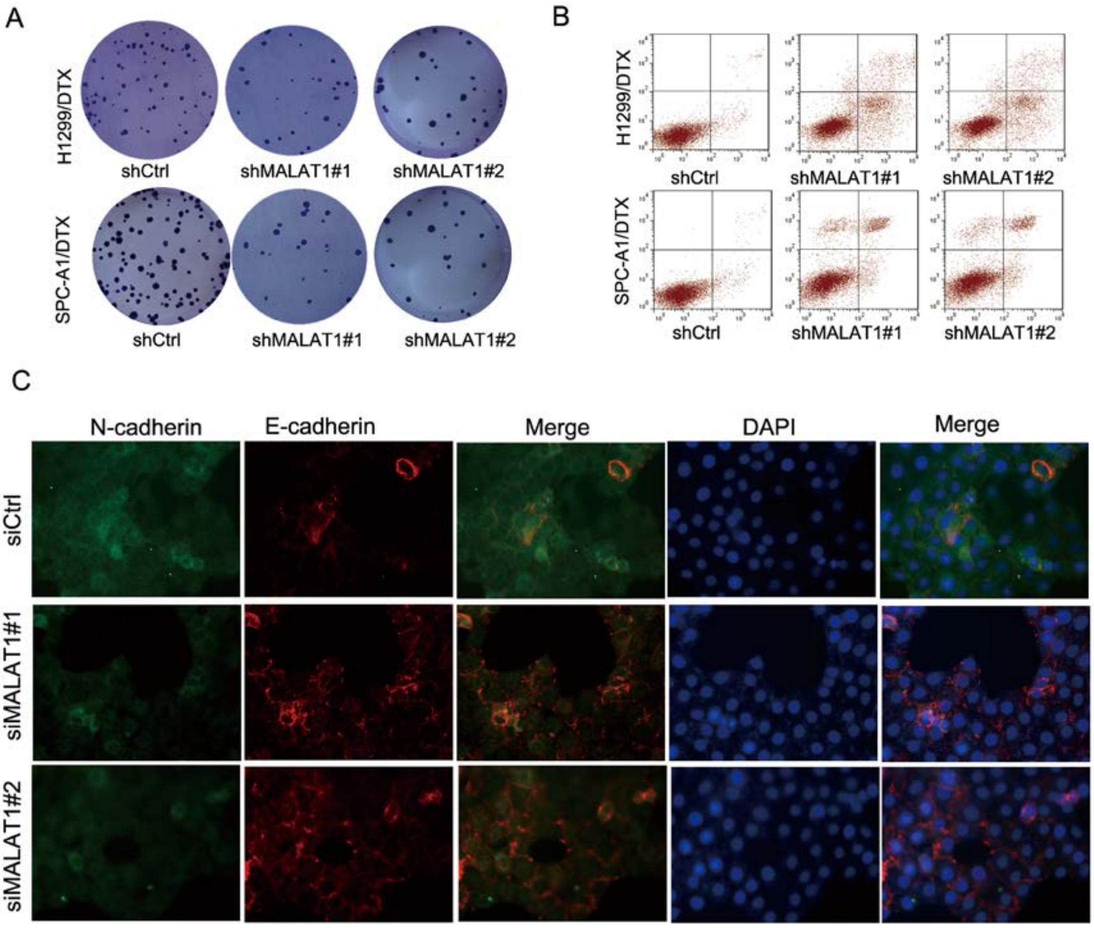

Supplementary Figure S5

A-C. Cell proliferation, apoptosis and EMT process were detected in SPC-A1/DTX and H1299/DTX cells transfected with shCtrl, shMALAT1#1 and shMALAT1#2.
