# Supplementary material for: TFAP2C-Activated MALAT1 Modulates the Chemoresistance of Docetaxel-Resistant Lung Adenocarcinoma Cells
Source: Mol Ther Nucleic Acids. 2019 Jan 18;14:567–82. doi: 10.1016/j.omtn.2019.01.005 (PMC6374643; doi:10.1016/j.omtn.2019.01.005)
Supplement: Document S2. Article plus Supplemental Information [file mmc3.pdf]

Molecular Therapy
Nucleic Acids
Original Article

TFAP2C-Activated MALAT1 Modulates the Chemoresistance of Docetaxel-Resistant Lung Adenocarcinoma Cells

Jing Chen,[1,2,6] Xiaobei Liu,[2,6] Yichen Xu,[2] Kai Zhang,[2] Jiayuan Huang,[3] Banzhou Pan,[3] Dongqin Chen,[4] Shiyun Cui,[5] Haizhu Song,[2] Rui Wang,[2] Xiaoyuan Chu,[2] Xiaoli Zhu,[1] and Longbang Chen[2]

[1]Department of Respiratory, Zhongda Hospital, Southeast University, Nanjing, China; [2]Department of Medical Oncology, Jinling Hospital, School of Medicine, Nanjing University, Nanjing, Jiangsu 210002, China; [3]Department of Medical Oncology, Jiangsu Cancer Hospital Affiliated to Nanjing Medical University, Jiangsu Institute of Cancer Research, Jiangsu, China; [4]Department of Medical Oncology, The First Affiliated Hospital of Soochow University, Suzhou, Jiangsu, China; [5]Department of Medical Oncology, The First Affiliated Hospital of Nanjing Medical University, Suzhou, Jiangsu, China

Chemoresistance remains a great obstacle in effective lung adenocarcinoma (LUAD) treatment. Previously, we verified the role of microRNA-200b (miR-200b) in the formation of docetaxel (DTX)-resistant LUAD cells. This study aims to investigate the mechanism underlying the low level of miR-200b in DTX-resistant LUAD cells. The real-time reverse transcription (RT^2) lncRNA PCR array system was applied to explore lncRNAs that potentially regulated miR-200b in DTX-resistant LUAD cells. Metastasis-associated lung adenocarcinoma transcript 1 (MALAT1) contributed to the low miR-200b level in DTX-resistant LUAD cells. Functional assays were conducted to determine the role of MALAT1 in regulating the growth and metastasis of parental and DTX-resistant LUAD cells. Investigation revealed the mechanism of the competing endogenous RNA (ceRNA) pathway. MALAT1 regulated miR-200b by acting as a ceRNA. MALAT1 modulated the sensitivity of LUAD cells to DTX. E2F transcription factor 3 (E2F3) and zinc-finger E-box binding homeobox 1 (ZEB1) were two targets of miR-200b and mediated the function of MALAT1 in DTX-resistant LUAD cells. Transcription factor AP-2 gamma (TFAP2C) and ZEB1 activated the MALAT1 transcription. In conclusion, TFAP2C-activated MALAT1 modulated the chemoresistance of LUAD cells by sponging miR-200b to upregulate E2F3 and ZEB1. Our findings may provide novel therapeutic targets and perspectives for LUAD treatment.

INTRODUCTION

Lung cancer is a kind of malignant tumor that has the high morbidity and mortality rates.[1,2] Among all kinds of lung cancers, non-small-cell lung cancer (NSCLC) accounts for approximately 85% of cases. Moreover, lung adenocarcinoma (LUAD) is most common subtype of NSCLC.[3] Although new technology regarding cancer treatment has developed and improved, the five-year survival rate of LUAD patients is still less than 10%.[4] In the early stage of chemotherapy, the NSCLC patients have relatively high sensitivity, while extensive metastasis and rapid progression result in chemoresistance.[5,6] Docetaxel (DTX) is a chemotherapeutic medicine that is quite useful for treating multiple cancers.[7] Despite the extensive use of DTX, the recurrence of LUAD is still common due to the chemoresistance.[8]

Based on genome sequencing technology, more than 98% of the human genomes are unable to code proteins.[9] Among these, long noncoding RNAs (lncRNAs) are a group of transcripts that are longer than 200 nucleotides.[10] An increasing number of studies have indicated that the aberrant expression of lncRNAs is closely associated with tumor sprouting, cancer cell growth, apoptosis, metastasis, and chemoresistance.[11–13] Many studies have demonstrated that lncRNAs affect the pathogenesis of human diseases by modulating gene expression via chromatin modification and transcriptional and post-transcriptional regulation.[14–17] Mechanistically, lncRNAs can serve as competing endogenous RNAs (ceRNAs) by sponging microRNA (miRNA) to upregulate mRNAs.[18,19] For instance, SNHG6-003 serves as a ceRNA to promote gastric cancer proliferation by sponging miR-1207-5p,[20] and lncRNA KCNQ1OT1 promotes cell proliferation and cisplatin resistance in tongue cancer via miR-211-5p-mediated Ezrin/Fak/Src signaling.[21] Increasing evidence indicates that lncRNAs can regulate the chemoresistance of various human cancers. For examples, lncRNA H19 can regulate the stemness and chemoresistance of colorectal cancer;[22] lncRNA LBCS inhibits chemoresistance of bladder cancer stem cells by epigenetically silencing SOX2.[23] Previously, we revealed that downregulation of microRNA-200b (miR-200b) contributes to the chemoresistance of DTX-resistant LUAD cells,[24] and we proved part of the regulatory mechanism that led to the abnormal expression of miR-200b.[25,26] However, it is unknown

Received 6 December 2018; accepted 9 January 2019;
https://doi.org/10.1016/j.omtn.2019.01.005.

[6]The authors contributed equally to this work.

Correspondence: Xiaoli Zhu, Department of Respiratory, Zhongda Hospital, Southeast University, Nanjing, China.
E-mail: zhuxiaoli62@163.com
Correspondence: Longbang Chen, Department of Medical Oncology, Jinling Hospital, School of Medicine, Nanjing University, Nanjing, Jiangsu 210002, China.
E-mail: chenlongbang@yeah.net

This is an open access article under the CC BY license (http://creativecommons.org/licenses/by/4.0/).

whether miR-200b can be regulated by lncRNA. Therefore, this study aims to investigate the upstream lncRNA of miR-200b in DTX-resistant LUAD cells.

We identified the lncRNA that could regulate miR-200b in DTX-resistant LUAD cell by screening with the real-time reverse transcription (RT^2) lncRNA PCR array system. Moreover, we identified the regulatory mode of Metastasis-associated lung adenocarcinoma transcript 1 (MALAT1) on miR-200b by performing mechanism experiments. *In vitro* and *in vivo* experiments were carried out in both parental and DTX-resistant LUAD cells to demonstrate the role of MALAT1 in regulating the DTX resistance of LUAD cells. Similarly, the targets of miR-200b were identified. Rescue assays were designed and applied to verify the role of the MALAT1/miR-200b/E2F transcription factor 3 (E2F3)/zinc-finger E-box binding homeobox 1 (ZEB1) axis in regulating LUAD chemoresistance. Finally, the upstream mechanism involved in the MALAT1/miR-200b/E2F3/ZEB1 axis was analyzed. In summary, this study revealed the mechanism and function of a novel molecular pathway in the chemoresistance of LUAD.

RESULTS

MALAT1 Expression Was Upregulated in DTX-Resistant LUAD Cells and Modulated miR-200b at the Post-transcriptional Level

An RT^2 lncRNA PCR array system was applied to explore potential lncRNAs involved in the modulation of miR-200b expression in DTX-resistant LUAD cells. As illustrated in Figure 1A and Figure S4A, three lncRNAs had a fold change $\geq$ 2.0 in SPC-A1/DTX, H1299/DTX, and A549/DTX cells compared with parental SPC-A1, H1299, and A549 cells. For further screening, we determined the expression level of miR-200b in two pairs of DTX-resistant LUAD cells and parental cells (Figure S1A); then, we used small interfering RNAs (siRNAs) to silence the endogenous levels of these three lncRNAs in SPC-A1/DTX and H1299/DTX cells (Figure S1B). qRT-PCR examination showed that only silencing of MALAT1 led to the significant upregulation of miR-200b (Figure 1B). To investigate the regulatory mode of MALAT1 on miR-200b, subcellular fractionation analyses and RNA fluorescence *in situ* hybridization (FISH) demonstrated that MALAT1 was distributed in both nucleus and cytosol (Figures 1C and 1D). Then, we found that knockdown of MALAT1 had no significant influence on the promoter activity of itself (Figure S1C). Furthermore, we assessed the levels of pri-miR-200b and pre-miR-200b in DTX-resistant LUAD cells transfected with MALAT1-specific siRNAs and found that MALAT1 knockdown didn't affect the levels of both pri-miR-200b and pre-miR-200b (Figure S1D), indicating that MALAT1 might regulate miR-200b in DTX-resistant LUAD cells at the post-transcription level. In general, lncRNAs regulate target genes by interacting with RNA binding proteins or by functioning as ceRNAs for specific miRNAs. An increasing number of studies have documented that lncRNAs can act as ceRNAs to sponge miRNAs through binding with miRNA response element (MRE).[27,28] miRNAs are known to exert functions by forming ribonucleoprotein complexes (RISCs), and Ago2 is the core component of RISCs. To test whether MALAT1 regulated miR-200b by acting as a ceRNA, RNA immunoprecipitation (RIP) assays were performed with SPC-A1 and SPC-A1/DTX cell extracts using anti-Ago2. As shown in Figure 1E and Figure S4E, MALAT1 and miR-200b were substantially enriched in the Ago2 immunoprecipitation compared with the negative control immunoglobulin G (IgG). Two binding sequences between MALAT1 and miR-200b were found from the online bioinformatics analysis (http://starbase.sysu.edu.cn/) (Figure 1F). To validate whether these two binding sequences were responsible for the interaction between MALAT1 and miR-200b, we mutated binding sequence 1 (Mut1) and binding sequence 2 (Mut2), respectively. Moreover, we mutated both binding sequence 1 and binding sequence 2 (Mut1/2). Then, we subcloned wild-type (WT) MALAT1 or mutant types of MALAT1 (Mut1, Mut2, or Mut1/2) into the pmirGLO vector. The results of luciferase reporter assays were performed in SPC-A1/DTX and HEK293T cells. The results showed that miR-200b mimics significantly decreased the luciferase activity of the WT reporter, Mut1 reporter, and Mut2 reporter, but not that of Mut1/2 reporter (Figure 1G), indicating that these two binding sequences were synergistically responsible for the interaction of MALAT1 and miR-200b. All these results revealed that MALAT1 might modulate miR-200b expression by acting as a ceRNA.

MALAT1 Enhanced the Chemoresistance of LUAD Cells by Affecting Cell Proliferation, Apoptosis, and Cell-Cycle Distribution

To investigate the effect of MALAT1 on the chemoresistance of LUAD, we transfected SPC-A1 and H1299 cells with MALAT1 expression vector or empty vector (Figure S1E). In contrast, we transfected SPC-A1/DTX and H1299/DTX cells with MALAT1-specific short hairpin RNAs (shRNAs) (shMALAT1#1 and shMALAT1#2) or control shRNA (shCtrl) (Figure S1F). In addition, the half maximal inhibitory concentration (IC_{50}) value of A549/DTX was higher that that of A549 (Figure S4B). Then, we detected the effect of MALAT1 overexpression or knockdown on IC_{50} in parental or DTX-resistant LUAD cells. The results of 3-(4,5-dimethylthiazol-2-yl)-2,5-diphenyl-tetrazolium bromide (MTT) assays revealed that MALAT1 overexpression significantly increased the IC_{50} value in parental cells, while MALAT1 knockdown led to the opposite effect in DTX-resistant LUAD cells (Figures 2A and 2B; Figures S4C and S4D). However, overexpression of mutant type of MALAT1 (MALAT1-MUT) and knockdown of MALAT1-MUT could not affect the IC_{50} of SPC-A1 and SPC-A1/DTX cells (Figure S4F). Then, we detected the effects of MALAT1 overexpression or knockdown on the biological processes of parental or DTX-resistant LUAD cells under the condition with or without DTX. To avoid an off-target effect, we tested the proliferation and apoptosis of DTX-resistant LUAD cells transfected with shMALAT1#1, shMALAT1#2, or shCtrl. The results showed that both shMALAT1#1 and shMALAT1#2 efficiently suppressed cell proliferation, promoted cell apoptosis, and reversed the epithelial-mesenchymal transition (EMT) process (Figure S5). Considering the better knockdown efficiency of shMALAT1#1, we chose it for all subsequent experiments. Furthermore, we found that MALAT1 overexpression increased the colony-forming ability in parental LUAD cells treated with or without DTX (0 or 10 μg/L). In contrast,

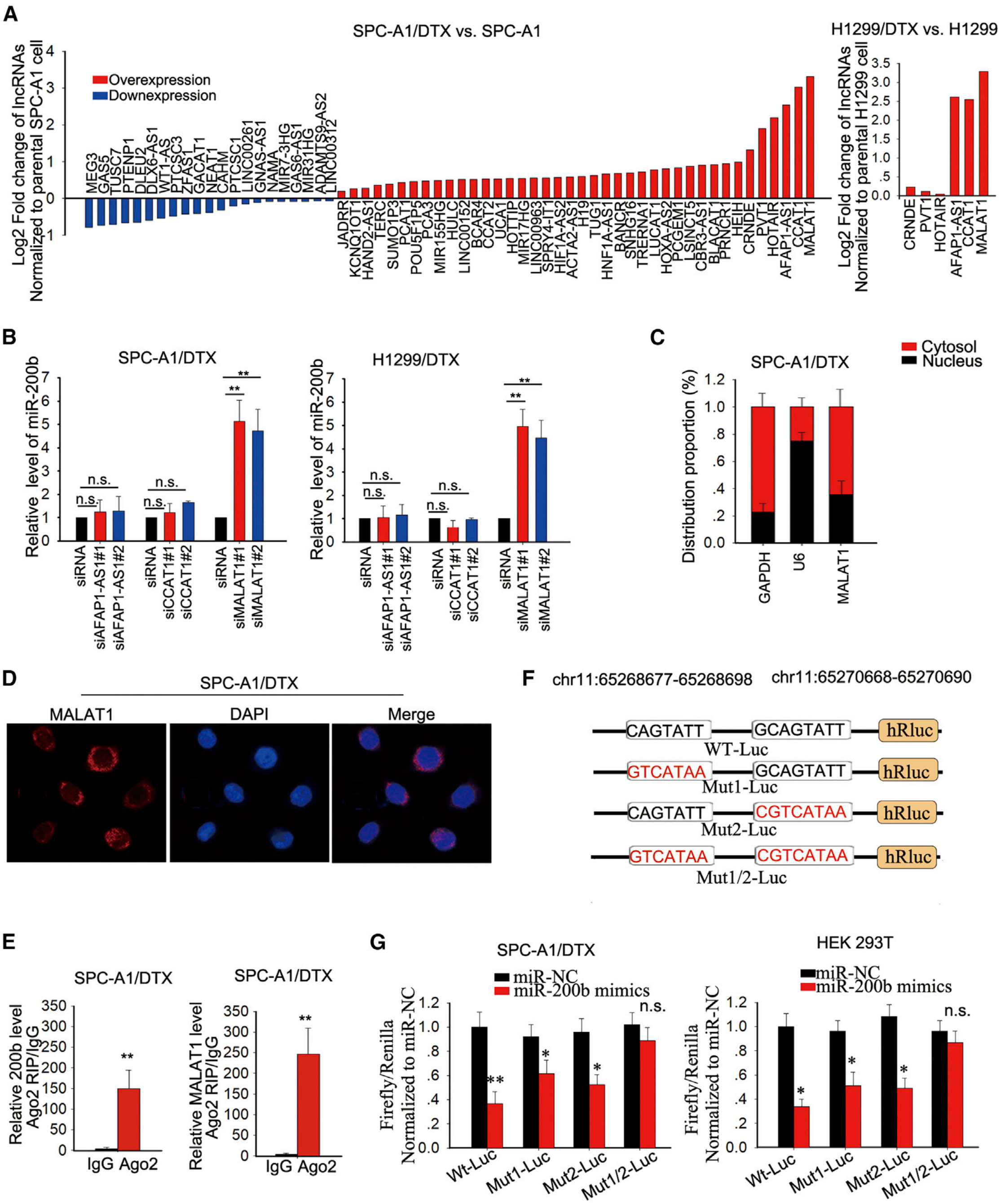

Figure 1. MALAT1 Expression Was Upregulated in Docetaxel-Resistant LUAD Cells and Modulated miR-200b at the Post-transcriptional Level

(A) An RT^2 lncRNA PCR array system was applied to measure the expression levels of lncRNAs that were potentially involved in the modulation of miR-200b expression in DTX-resistant LUAD cells. The results were compared with those from parental SPC-A1 and H1299 cells. (B) The effect of the three lncRNAs on the miR-200b expression level was detected by qRT-PCR in both SPC-A1/DTX and H1299/DTX cells. (C) Subcellular fractionation of SPC-A1 cell to determine the cellular location of MALAT1. (D) RNA FISH was used to identify the MALAT1 location. (E) RIP experiments revealed the enrichment of MALAT1 and miR-200b in the Ago2 immunoprecipitation compared with the control IgG precipitation. (F) Two binding sites of MALAT1 to miR-200b were predicted. (G) Two binding sites were separately mutated (Mut1 and Mut2) or simultaneously mutated (Mut1/2) and were constructed into different vectors. The wild-type (WT) vector and three mutant-type vectors were cotransfected into SPC-A1/DTX and HEK293T cells, together with miR-200b mimics or miR-NC. The reporter vectors were normalized to Renilla luciferase vector. $^*p < 0.05$, $^{**}p < 0.01$; n.s., no significance.

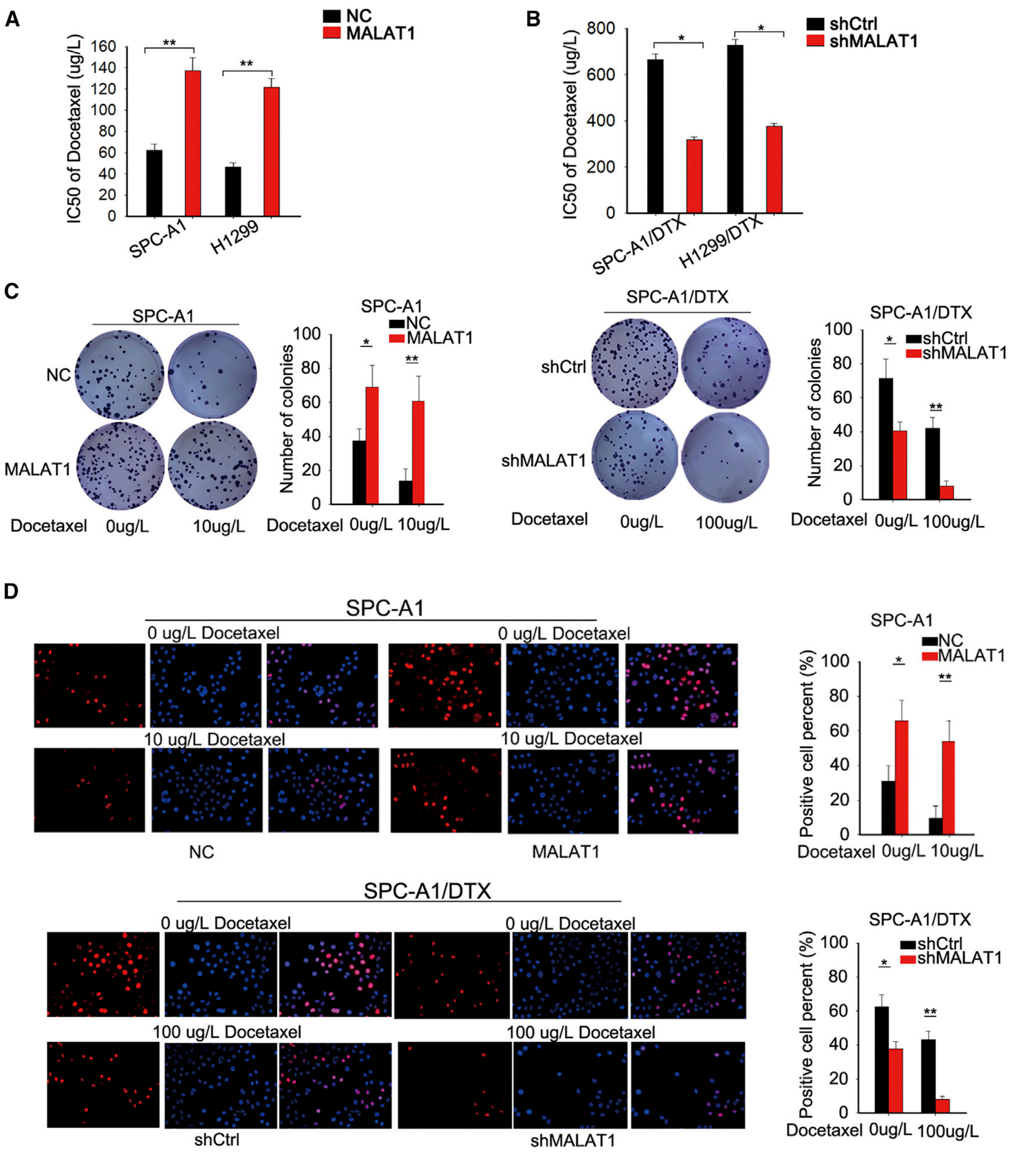

Figure 2. MALAT1 Enhanced the Chemoresistance of LUAD Cells by Affecting Cell Proliferation

(A) The effect of MALAT1 overexpression on the IC_{50} value of SPC-A1 and H1299 cells was measured by MTT assays. (B) The effect of MALAT1 knockdown on SPC-A1/DTX and H1299/DTC cell sensitivity to DTX was detected by MTT assays. (C) Representative colony formation results in SPC-A1 cells transfected with MALAT1 expression vector and SPC-A1/DTX cells transfected with MALAT1 shRNA. (D) EdU proliferation assay in MALAT1-overexpressed SPC-A1 cells or MALAT1-downregulated SPC-A1/DTX cells. Scale bar is 200 μm. $*p < 0.05$, $**p < 0.01$.

knockdown of MALAT1 significantly inhibited the colony-forming ability of DTX-resistant LUAD cells treated with or without DTX (0 or 100 μg/L) (Figure 2C; Figure S2A). Consistently, similar positive effects of MALAT1 overexpression on the proliferation of parental cells, as well as the negative effect of MALAT1 knockdown on the proliferation of DTX-resistant LUAD cells, were validated by 5-ethynyl-2′-deoxyuridine (EdU) proliferation assays (Figure 2D; Figure S3A). Furthermore, we applied flow cytometry analyses to determine whether MALAT1 regulated cell proliferation by affecting cell apoptosis and cell-cycle distribution. The results indicated that overexpression of MALAT1 led to decreased apoptosis rate in parental cells, while MALAT1 knockdown increased the apoptosis rate in

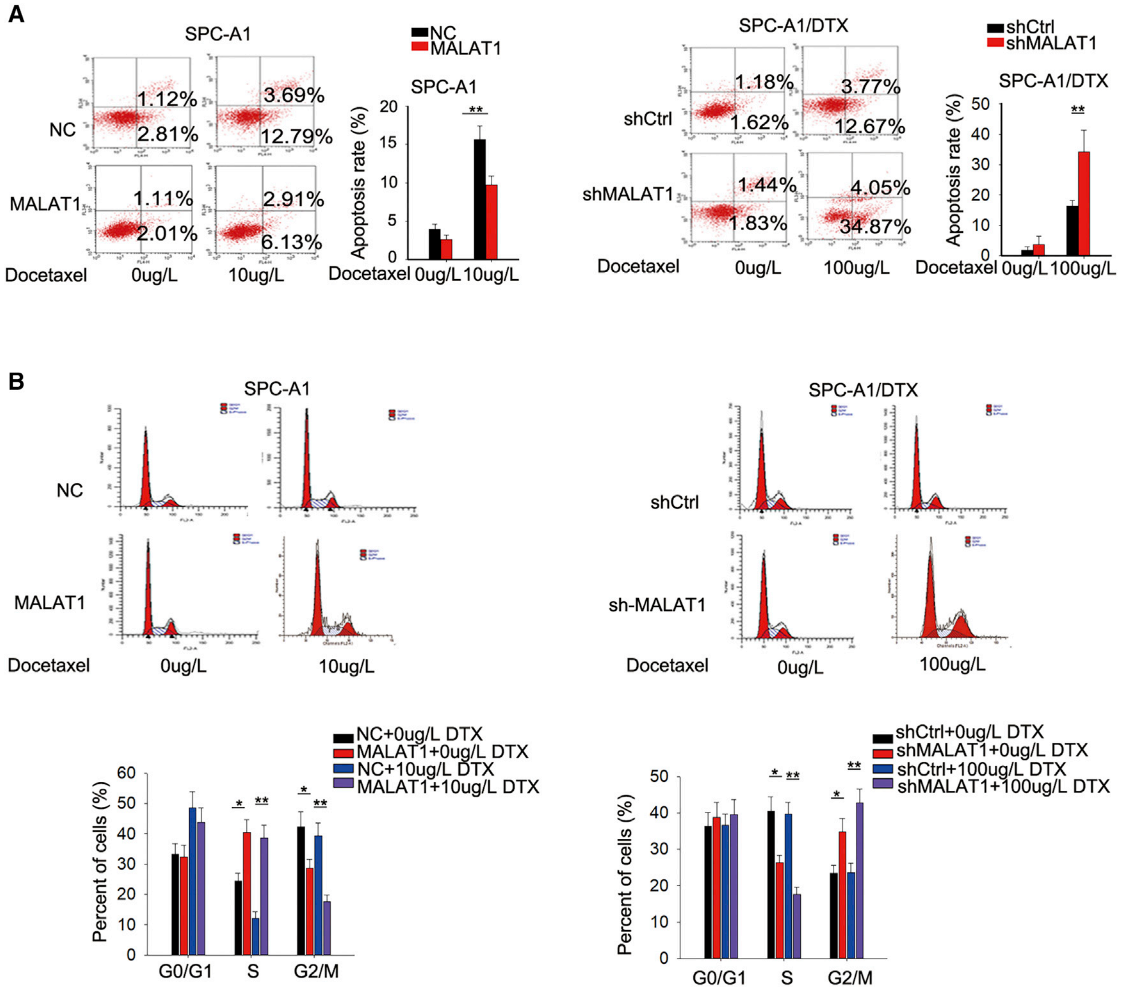

Figure 3. Dysregulated MALAT1 Influenced Cell Apoptosis Rate and Cell-Cycle Distribution

(A) Flow cytometric analysis of apoptosis in SPC-A1 cells transfected with pcDNA3.1 empty vector or pcDNA3.1/MALAT1 with or without treatment of DTX or in SPC-A1/DTX cells transfected with shCtrl or shMALAT1 with or without treatment of DTX. (B) Flow cytometric analysis of cell cycle distribution in indicated cells. *$p < 0.05$, **$p < 0.01$.

DTX-resistant LUAD cells (Figure 3A; Figure S2B). Overexpression of MALAT1 promoted the cell-cycle progress in parental cells, while knockdown of MALAT1 induced cell-cycle arrest at the G0/G1 phase in DTX-resistant LUAD cells (Figure 3B; Figure S2C). In addition, the expression levels of proteins related to cell-cycle progress and apoptosis were tested by western blot analysis. As illustrated in Figure S4G, the levels of apoptosis-related proteins (cleaved caspase-3 and cleaved caspase-6) were efficiently decreased in parental cell by the overexpression of MALAT1. In contrast, the levels were increased by the knockdown of MALAT1 in DTX-resistant LUAD cells. Moreover, the levels of cell-cycle-related proteins were increased by MALAT1 overexpression but were decreased by MALAT1 knockdown (Figure S4H). The changes in the protein levels were more significant when cells were treated with cisplatin. These findings suggested that MALAT1 enhanced the resistance of both parental and DTX-resistant LUAD cells to DTX by increasing cell proliferation ability, reducing apoptosis, and promoting cell-cycle progression.

MALAT1 Was Involved in the EMT Process of Parental and DTX-Resistant LUAD Cells

EMT phenotype is a crucial biological process that has been demonstrated to be closely associated with the LUAD chemoresistance. In our study, we detected the role of MALAT1 in the EMT progress. As presented in Figure 4A, the morphology of DTX-resistant cells was distinctly different from that of parental cells because of loss of cell polarity, intercellular adhesion, and formation of pseudopodia. To determine whether MALAT1 promoted DTX-induced EMT phenotype, we measured the expression levels of EMT markers in both parental and DTX-resistant cells. As a result, MALAT1 overexpression decreased the protein levels of epithelial markers (E-cadherin and β-catenin) and increased the levels of mesenchymal markers (N-cadherin and Vimentin) in parental cells. In contrast, knockdown of MALAT1 in DTX-resistant cells led to the opposite results (Figure 4B; Figure S3C). Moreover, the results of immunofluorescence staining were consistent with that of western blot (Figure 4C;

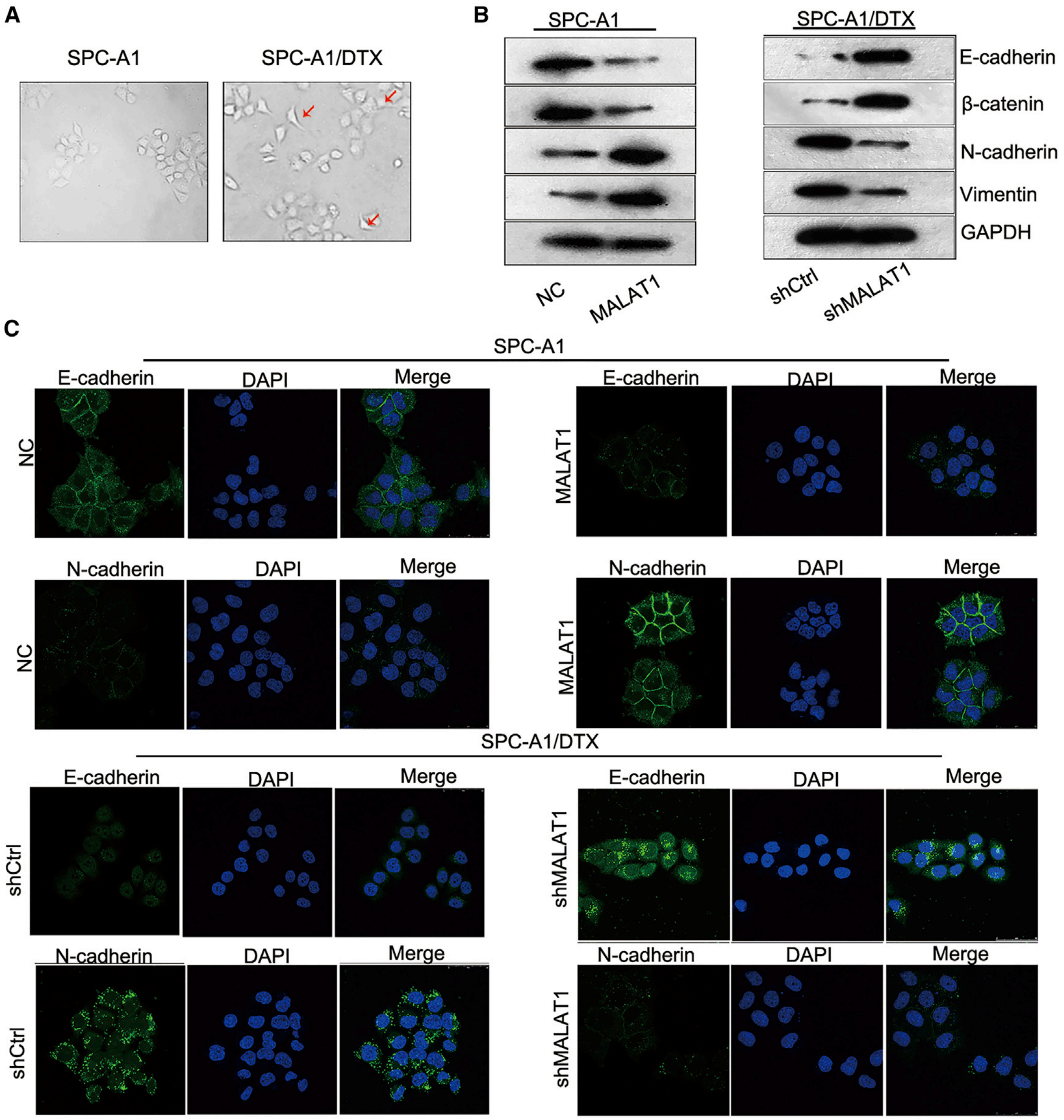

Figure 4. MALAT1 Was Involved in the EMT Process of Parental and DTX-Resistant LUAD Cells

(A) Morphology of SPC-A1 and SPC-A1/DTX cells. (B) Protein levels of E-cadherin and N-cadherin in SPC-A1 cells transfected with pcDNA3.1 or pcDNA3.1/MALAT1 and SPC-A1/DTX cells transfected with shRNA or shMALAT1. (C) The levels of E-cadherin and N-cadherin were determined in indicated cells by immunofluorescence staining. Scale bar is 200 μm. *p < 0.05, **p < 0.01.

Figure S3B), which further demonstrated the positive effect of MALAT1 on EMT progress.

Knockdown of MALAT1 Suppressed the Growth and Metastasis of DTX-Resistant LUAD Cells *In Vivo*

To verify the results of *in vitro* experiments, *in vivo* experiments were carried out in SPC-A1/DTX cells. At first, we stably transfected SPC-A1/DTX cells with shMALAT1, while cells transfected with shCtrl were used as negative controls. Then, cells were injected into nude mice to assess the effect of MALAT1 knockdown on cell growth *in vivo*. Without DTX treatment, tumors derived from SPC-A1/DTX cells stably transfected with shMALAT1 grew slower than those derived from control cells, and this phenomenon was more significant in tumors treated with DTX (Figures 5A–5C). Compared with tumors derived from control cells, those from SPC-A1/DTX cells stably transfected with shMALAT1 exhibited reduced Ki-67 and proliferating cell nuclear antigen (PCNA) positivity (Figure 5D). The inhibition of MALAT1 knockdown on Ki-67 and PCNA positivity was more

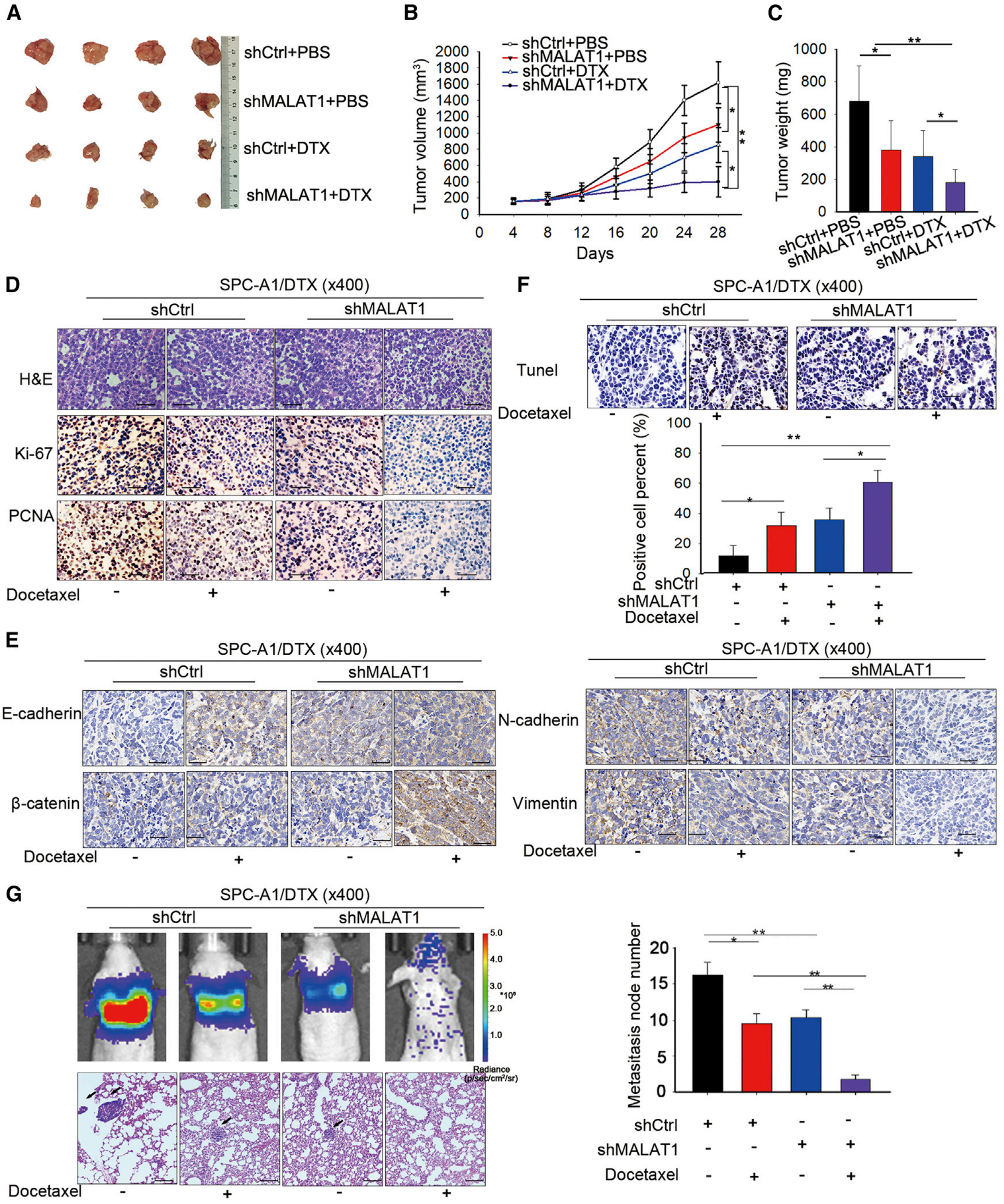

Figure 5. Knockdown of MALAT1 Suppressed the Growth and Metastasis of DTX-Resistant LUAD Cells *In Vivo*

(A) Tumors derived from LUAD cells transfected with shMALAT1 or shCtrl were measured under a condition with PBS or DTX. (B) Tumor volume was measured. (C) Tumor weight was calculated. (E) The tumor sections were subjected to immunohistochemical (IHC) staining with antibodies against E-cadherin and N-cadherin. (F) TUNEL staining of tumor tissues was applied to detect apoptosis. Scale bar, 50 μm. (G) A luciferase xenograft mouse model was created to demonstrate the effect of MALAT1 on tumor metastasis *in vivo*; the entire lung was visualized. Scale bar, 100 μm. $^{*}p < 0.05$, $^{**}p < 0.01$.

efficient when treating with DTX. Moreover, the increased levels of E-cadherin and β-catenin, as well as the decreased levels of N-cadherin and Vimentin, were detected in tumors derived from SPC-A1/DTX cells stably transfected with shMALAT1 (Figure 5E). In addition, transferase dUTP nick-end labeling (TUNEL) staining results revealed that tumors formed from SPC-A1/DTX cells stably

transfected with shMALAT1 presented with a significantly increased apoptosis rate (Figure 5F). Moreover, a luciferase xenograft mouse model was established to determine whether MALAT1 expression affects tumor metastasis *in vivo*. Luciferase SPC-A1/DTX cells stably transfected with lentiviral vector (lv)-shRNA targeting MALAT1 were subcutaneously injected into the tail veins of nude mice. As shown in Figure 5G, ectopic MALAT1 knockdown reduced the number of metastatic cells compared with the control group; the result was confirmed by testing H&E-stained lung sections. In all experimental results shown in Figure 5, the effect of MALAT1 knockdown on tumor growth was more significant when treating with DTX.

MALAT1 Promoted LUAD Chemoresistance by Sponging miR-200b to Increase the Expression of E2F3 and ZEB1

The preceding findings suggested that MALAT1 might regulate miR-200b by acting as a ceRNA. Thus, we found the possible targets of miR-200b using three bioinformatics prediction tools: TargetScan (http://www.targetscan.org/), RNA22 (http://www.rna-seqblog.com/rna22-version-2-0-mirna-mre-predictions/), and PicTar (http://pictar.mdc-berlin.de). There were 23 mRNAs that might interact with miR-200b (Figure 6A). We then assessed the expression levels of these mRNAs in parental and DTX-resistant cells. The results showed that E2F3 and ZEB1 were upregulated in DTX-resistant LUAD cells (Figure 6B). Furthermore, we found that the levels of E2F3 and ZEB1 were positively regulated by MALAT1, while miR-200b mimics or inhibitors partially attenuated the effects of MALAT1 overexpression or knockdown on the expression levels of E2F3 and ZEB1 (Figures 6C and 6D; Figure S4I), indicating that MALAT1 regulates E2F3 and ZEB1 expression in a miR-200b-dependent manner. To verify the competitive relationship between MALAT1 and E2F3/ZEB1, we performed RIP assays and found the existence of both E2F3 and ZEB1 in the Ago2 complex (Figure 6E; Figure S4J, upper), indicating that MALAT1 competes with E2F3/ZEB1 to bind miR-200b. In addition, we applied dual-luciferase reporter assays and observed that miR-200b mimics inhibited firefly luciferase activity of WT ZEB1 or E2F3 3′ UTRs, but not that of mutant type; this effect was abolished by the introduction of MALAT1 (Figure 6F). Then, we performed rescue assays to demonstrate the role of the MALAT1/miR-200b/E2F3/ZEB1 axis in regulating the chemoresistance of parental and DTX-resistant LUAD cells. As presented in Figure 7A, the increased IC_{50} value in parental cell was partially attenuated by the overexpression of miR-200b or the knockdown of E2F3. In DTX-resistant cells, the IC_{50} value decreased by MALAT1 was rescued by the downregulation of miR-200b or the overexpression of E2F3 (Figure 7B). Furthermore, knockdown of MALAT1 weakened proliferative ability and increased apoptosis rate, which could be abolished largely by the introduction of miR-200b inhibitors or E2F3, but not ZEB1 (Figures 7C and 7D). In addition, overexpression of MALAT1 promoted EMT progress in parental LUAD cells, while this function was mediated by miR-200b mimics or shRNA specifically targeted to E2F3 (sh-E2F3) (Figure 7E). Introduction of miR-200b inhibitors or E2F3 expression vector reversed the EMT progress inhibited by MALAT1 knockdown. Therefore, we confirmed that miR-200b and E2F3 are involved in MALAT1-mediated chemoresistance in LUAD.

TFAP2C and ZEB1 Transcriptionally Activated MALAT1 in DTX-Resistant LUAD Cells

Although MALAT1 was upregulated in DTX-resistant LUAD cells, the mechanism associated with MALAT1 upregulation has not been properly investigated. It has been reported that lncRNAs can be activated by their upstream transcription factors in human malignant tumors.[29] To investigate the transcription factors that were responsible for the upregulation of MALAT1, we performed a pull-down experiment in the nuclear extract using the biotin-labeled MALAT1 promoter. Nuclear proteins of LUAD cells were incubated and pulled down by biotin-labeled MALAT1 promoter. One-shot mass spectrometry analyses were then performed to analyze the purified nuclear proteins. As shown in Figure 8A, 8 transcription factors (TFs) were found to be enriched in SPC-A1/DTX group. Based on online bioinformatics analysis (http://genome.ucsc.edu/index.html), we found the binding region of transcription factor AP-2 gamma (TFAP2C) on the promoters of MALAT1 and ZEB1 (> hg19 dna range = chr11:65264902-65265225;strand = + and > hg19 dna range = chr10:31607523-31608038;strand = +), as well as the binding region of ZEB1 on the promoter of MALAT1 (> hg19 dna range = chr11:65263903-65264418;strand = +) (Figure 8B). Then, we measured the expression level of TFAP2C in two pairs of DTX-resistance LUAD cell lines and their parental cell lines. As illustrated in Figure 8C, the mRNA and protein levels of TFAP2C were increased in the DTX-resistant cells. To determine the roles of TFAP2C and ZEB1 on the expression level of MALAT1, we measured the expression level of MALAT1 in DTX-resistant LUAD cells transfected with sh/TFAP2C and sh-ZEB1. As shown in Figure 8D, knockdown of TFAP2C or ZEB1 efficiently reduced the level of MALAT1 both in SPC-A1/DTX and H1299/DTX cells. In addition, knockdown of TFAP2C could reduce the mRNA and protein levels of ZEB1 (Figure 8E). Using chromatin immunoprecipitation analysis, we found the affinity of TFAP2C to the MALAT1 promoter and the ZEB1 promoter (Figure 8F; Figure S4J, bottom); the GAPDH promoter was used as a positive control (Figure 8G). These findings revealed that TFAP2C and ZEB1 transcriptionally activated MALAT1, thereby contributing to the upregulating of MALAT1 in DTX-resistant LUAD cell lines. In addition, TFAP2C could transcriptionally activate ZEB1 expression, forming a feedback loop, which strengthening the function of MALAT1 in the chemoresistance of LUAD cells.

DISCUSSION

With the increasing identification of noncoding RNAs, the physiology and pathology of human cancers have been better understood.[30] The dysregulation of lncRNAs is closely related to the tumorigenesis of various cancer types, which provides new perspectives for the treatment of malignant cancers. Increasingly, reports have pointed out that some lncRNAs serve as a scaffold of protein complex to promote or inhibit gene expression at the transcription level,[31,32] but lncRNAs can also act as a mediator in ceRNA pathway to regulate tumorigenesis.[33–36]

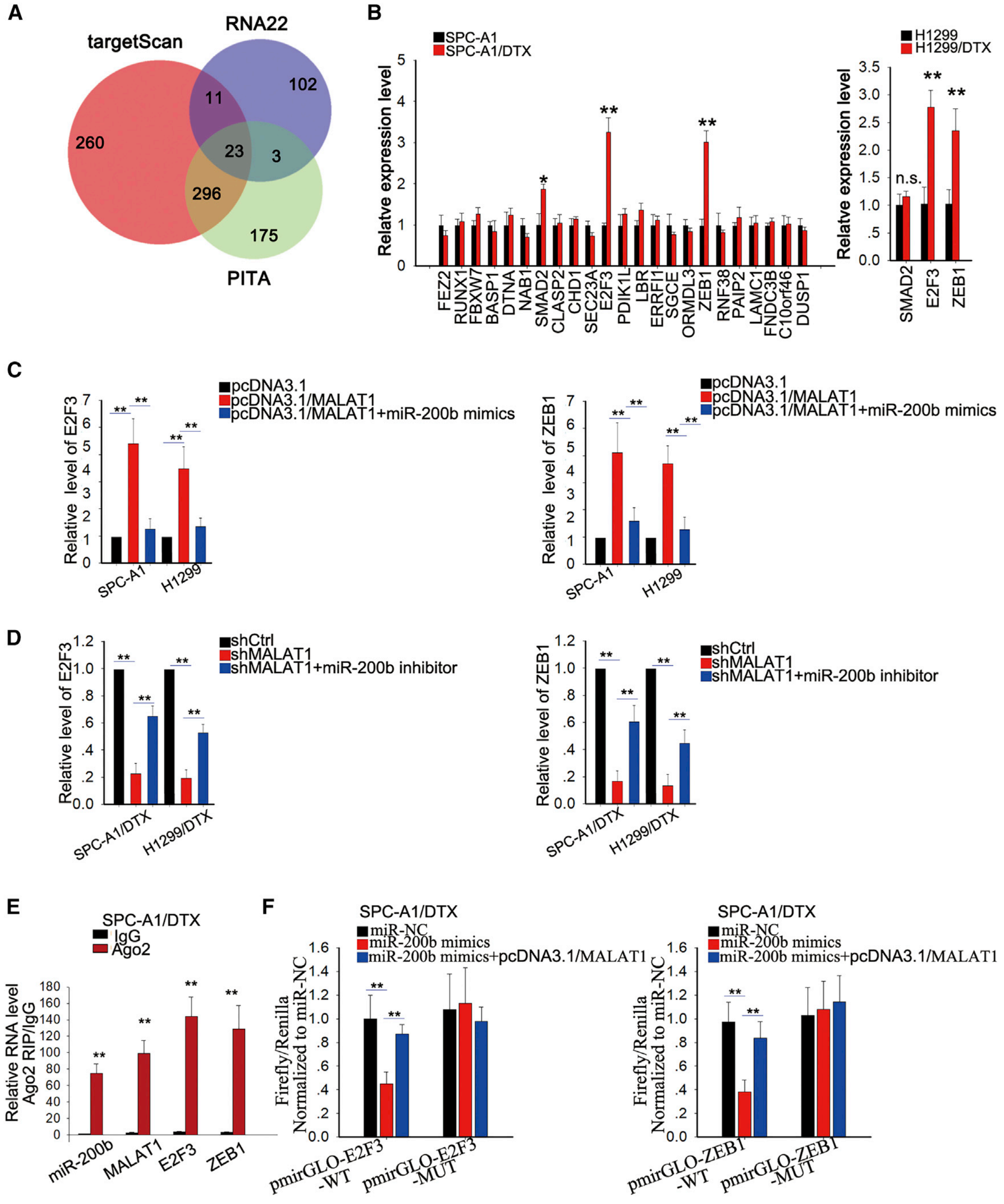

Figure 6. MALAT1 Promoted LUAD Chemoresistance by Sponging miR-200b to Increase the Expression of E2F3 and ZEB1

(A) The overlapping mRNAs from three online analysis tools, TargetScan (http://www.targetscan.org/), RNA22 (http://www.rna-seqblog.com/rna22-version-2-0-mirna-mre-predictions/), and PicTar (http://pictar.mdc-berlin.de), were identified with a Venn diagram. (B) The level of these mRNAs in SPC-A1/DTX and SPC-A1 cells was measured and validated in H1299/DTX and H1299 cells. (C) The mRNA levels of E2F3 and ZEB1 in parental cells transfected with pcDNA-MALAT1 or co-transfected with miR-200b mimics. (D) The mRNA levels of E2F3 and ZEB1 in DTX-resistant cells transfected with sh-MALAT1 or co-transfected with miR-200b inhibitor. (E) RIP assays were performed to determine the existence of MALAT1 and E2F3/ZEB1 in the Ago2 complex. (F) Dual-luciferase reporter assays were performed to confirm the association of miR-200b, MALAT1, and ZEB1 or E2F3. *p < 0.05, **p < 0.01; n.s., no significance.

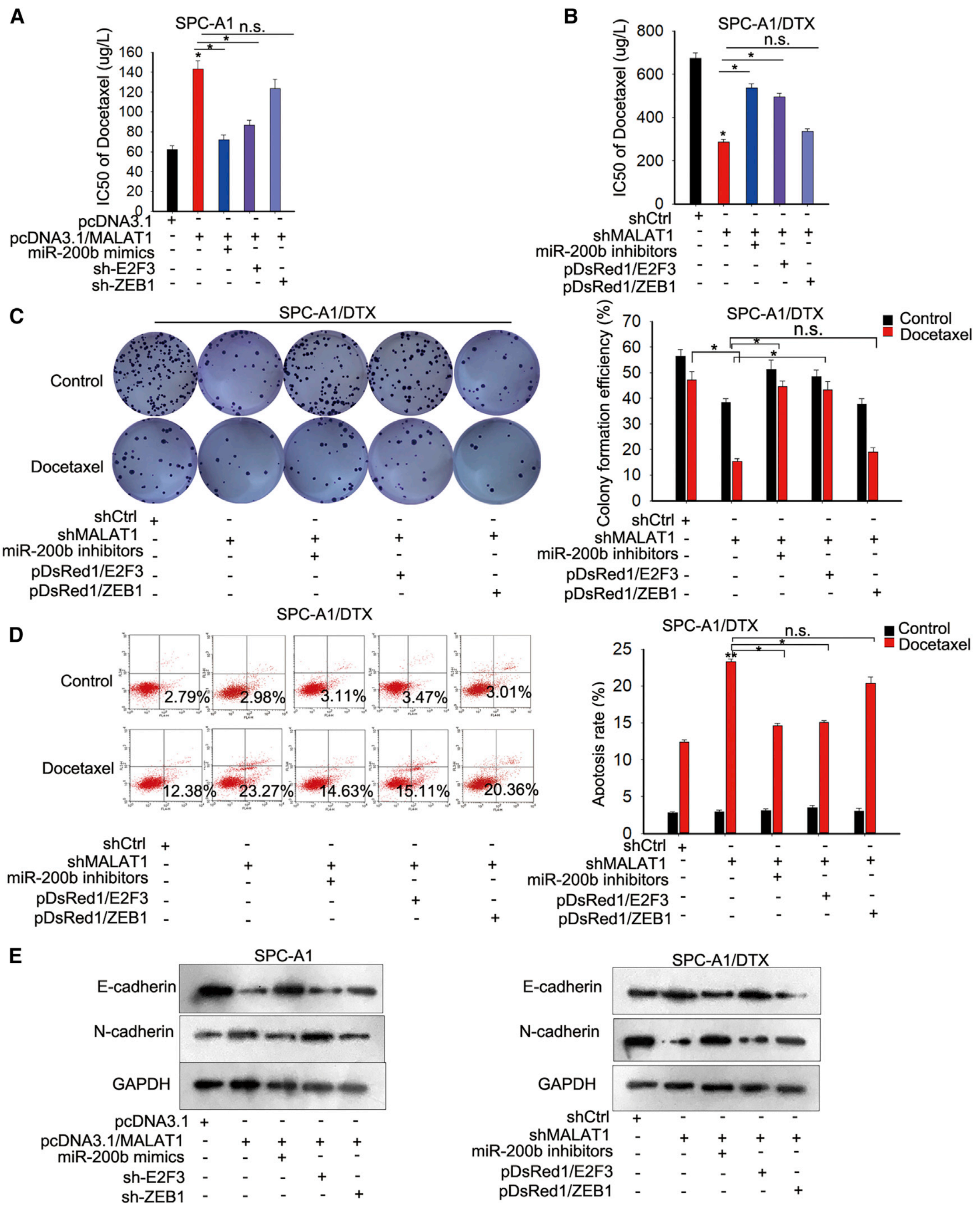

Figure 7. The Biological Role of MALAT1 in LUAD Cells Was Mediated by miR-200b and E2F3

(A) MTT assays were performed to determine the DTX sensitivity of SPC-A1 cells cotransfected with MALAT1 and miR-200b mimics or sh-E2F3 or sh-ZEB1. (B) MTT assays were performed to determine the DTX sensitivity of SPC-A1/DTX cells cotransfected with shMALAT1 and miR-200b inhibitors or E2F3 or ZEB1. (C) Colony formation assays were used to assess the proliferative ability of SPC-A1/DTX cells cotransfected with shMALAT1 and miR-200b inhibitors or E2F3/ZEB1 overexpression vector. (D) Flow cytometric analysis was used to measure the apoptosis rate of SPC-A1/DTX cells cotransfected with shMALAT1 and miR-200b inhibitors or E2F3/ZEB1 expression vector. (E) Western blot assays were used to measure EMT markers in SPC-A1/DTX cells cotransfected with shMALAT1 and miR-200b inhibitors or E2F3/ZEB1 expression vector. $^{*}p < 0.05$, $^{**}p < 0.01$; n.s., no significance.

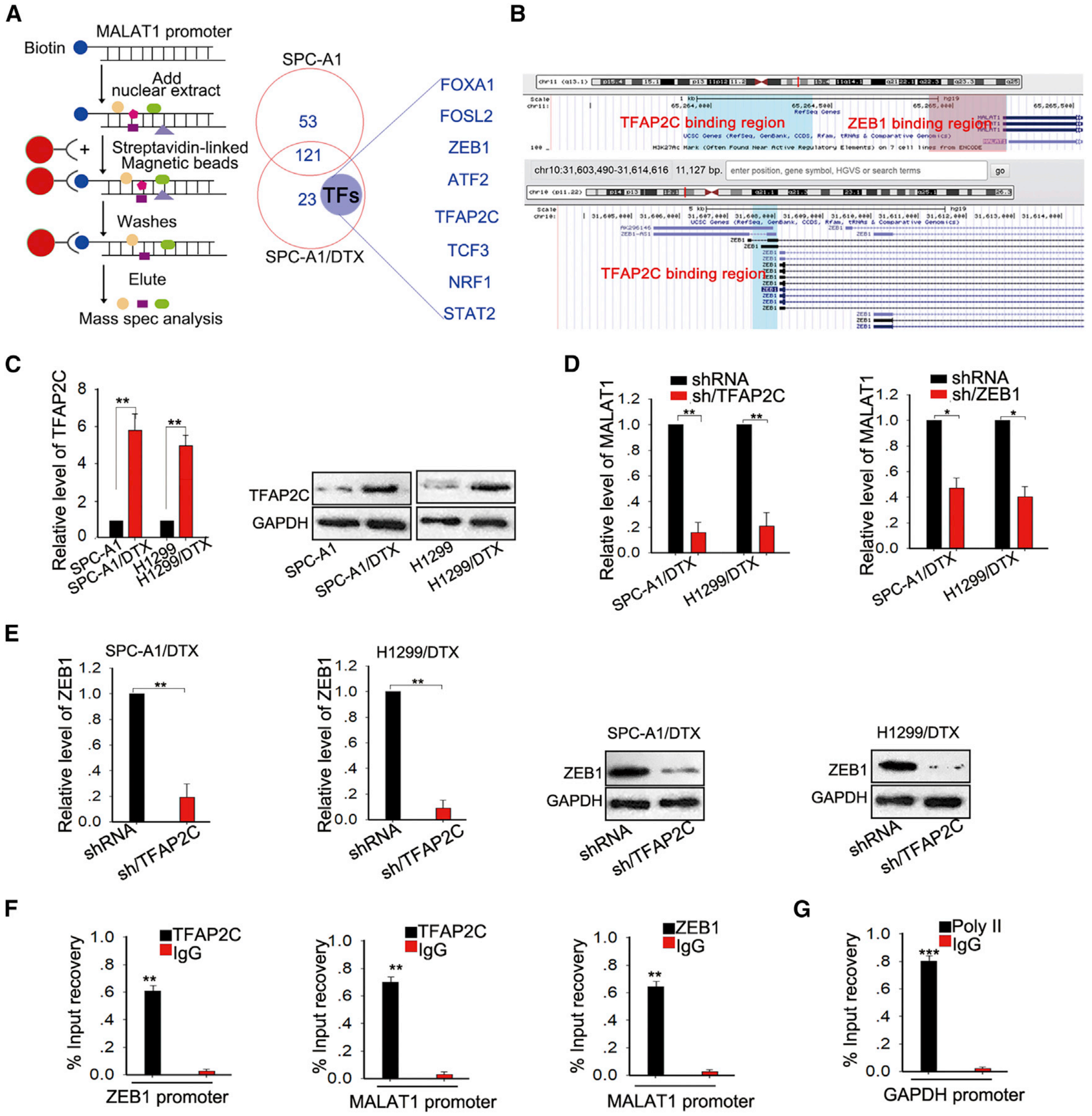

Figure 8. TFAP2C and ZEB1 Transcriptionally Activated MALAT1 in LUAD Cells

(A) The biotin-labeled MALAT1 promoter was mixed with the nuclear extract separated from SPC-A1 or SPC-A1/DTX. The eluted proteins were then analyzed using mass spectrometry methods. (B) The binding region of TFAP2C on the promoter of MALAT1 and ZEB1 (> hg19 dna range = chr11:65264902-65265225;strand = + and > hg19 dna range = chr10:31607523-31608038;strand = +), as well as the binding region of ZEB1 on the promoter of MALAT1 (> hg19 dna range = chr11:65263903-65264418;strand = +). (C) The expression level of TFAP2C in DTX-resistant LUAD cells and the corresponding parental cells was measured by qRT-PCR and western blot. (D) MALAT1 expression was analyzed by qRT-PCR methods in SPC-A1/DTX or H1299/DTX cells transfected with sh/TFAP2C or sh-ZEB1 for 48 h. (E) The effect of TFAP2C knockdown on the expression level of ZEB1 was measured by qRT-PCR and western blot assays. (F) ChIP assay using anti-ZEB1 or anti-TFAP2C or anti-IgG antibodies were performed to determine the affinity of ZEB1 and TFAP2C on the MALAT1 promoter and the affinity of TFAP2C on the ZEB1 promoter in SPC-A1/DTX cells. (G) GAPDH was used as a positive control in ChIP assay. *p < 0.05, **p < 0.001, ***p < 0.001.

Given that the pivotal role of lncRNAs as the regulators of gene expression, they have been reported to play significant roles in LUAD.[37,38] In present study, we discovered that MALAT1 regulated the expression levels of ZEB1 and E2F3 by modulating miR-200b so as to regulate cell chemoresistance. In addition, we identified the effect of a transcription factor, TFAP2C, on the transcription activation of MALAT1 and ZEB1; ZEB1 could also transcriptionally regulate the expression of MALAT1, forming a positive feedback loop. miR-200b has been demonstrated to be an important tumor suppressor.[39,40] To identify the lncRNA involved in miR-200b-mediated chemoresistance in LUAD, we applied the RT^2 lncRNA PCR system and found that MALAT1 had an influence on the expression level of

miR-200b, presenting a negative expression correlation with miR-200b. Former studies have illustrated that MALAT1 upregulation promoted tumorigenesis in numerous cancers, including in esophageal carcinoma,[41] prostate cancer,[42] hepatocellular carcinoma,[43] and lung cancer.[44] Although the role of MALAT1 has been explored in diverse cancers, its biological function in the DTX resistance of LUAD still needs to be elucidated.

Our study uncovered that inhibiting MALAT1 expression could sensitize LUAD cells to DTX, suppressing proliferation and EMT phenotype of DTX-resistant LUAD cells. What's more, rescue assays demonstrated that knockdown of MALAT1 inhibited LUAD cell proliferation through the miR-200b/E2F3 axis, while knockdown of MALAT1 inhibited EMT progression through the miR-200b/ZEB1 axis. Furthermore, through subcellular fractionation followed by qRT-PCR analysis, as well as the FISH localization, we discovered that MALAT1 was expressed in both nucleus and cytoplasm. To confirm whether MALAT1 regulated miR-200b expression on the transcription level or on the post-transcription level, we established a luciferase reporter system and found that the dysregulation of MALAT1 had no effect on the miR-200b promoter, indicating that MALAT1 could regulate miR-200b on the post-transcription level. Research has shown that lncRNA could regulate miRNAs through the ceRNA mechanism, in which lncRNA acts as the sponge of miRNA to competitively bind to miRNA through complementary base pairing, weakening the interaction between miRNA and its target mRNA.[45,46] Using bioinformatics tools, two potential binding sites on MALAT1 with miR-200b were identified. Then, we proved the interaction between MALAT1 and miR-200b through the luciferase reporter assay. In addition, through the observation of enriched expression of MALAT1 and miR-200b in Ago2 immunoprecipitation, the interaction between MALAT1 and miR-200b was confirmed. Subsequently, function assays *in vitro* validated the effect of MALAT1 and miR-200b on cell resistance to DTX, cell proliferation, apoptosis, invasion, migration, and EMT progression. Likewise, we certified the interaction of miR-200b with ZEB1 and E2F3 through the same methods. Altogether, our study revealed that MALAT1 weakened the inhibition of miR-200b on ZEB1 and E2F3 through the ceRNA network, promoting cell resistance to DTX in LUAD. In addition, a pull-down assay combined with mass spectrometry revealed that TFAP2C is potentially correlated with the transcription of both MALAT1 and ZEB1. We also found that ZEB1 could induce the transcription of MALAT1, which formed a positive feedback loop of ZEB1/MALAT1/miR-200b/ZEB1 and promoted the upregulation of MALAT1 in DTX-resistant LUAD cells.

In conclusion, MALAT1 promoted LUAD chemoresistance through miR-200b. In cytoplasm, MALAT1 acted as a molecular sponge for miR-200b to weaken the binding of miR-200b with E2F3 and ZEB1 mRNA, upregulating their expressions and promoting LUAD cell proliferation, invasion, migration, and EMT progression, as well as chemoresistance. TFAP2C and MALAT1 were found to contribute biologically to chemoresistance in LUAD, providing novel therapeutic targets and perspectives for LUAD treatment.

MATERIALS AND METHODS

Cell Lines and Culture

SPC-A1 and H1299 cell lines were obtained from the Tumor Cell Bank of the Chinese Academy of Medical Science (Shanghai, China). Cells were cultivated in RPMI 1640 medium (Invitrogen, Carlsbad, CA, USA), which was supplemented with 10% fetal bovine serum (FBS) (Invitrogen), ampicillin (100 U/mL), and streptomycin (100 μg/mL) at 37°C in humidified air with 5% CO_2. DTX-resistant LUAD cells (SPC-A1/DTX and H1299/DTX) were established from parental SPC-A1 and H1299 cells and were preserved in 50 μg/L DTX (final concentration).

Cell Transfection

siRNAs for MALAT1 were defined as siMALAT1#1 and siMALAT1#2, respectively. All siRNA and shRNA sequences are listed in Table S1. E2F3 and ZEB1 cDNA was amplified and cloned into the pDsRed1 expression vector. MALAT1 cDNA containing the miR-200b binding site was amplified and cloned into the pcDNA3.1 expression vector. siRNAs and plasmids were transfected into LUAD cells by applying Lipofectamine 2000 (Invitrogen) according to the manufacturer's instructions.

Construction of Stable MALAT1-Silenced Cell Lines

The expression level of MALAT1 was stably inhibited by transfecting with desired shRNA vectors (shMALAT1#1 and shMALAT1#2) or shCtrl and was screened with puromycin (2 μg/mL) for 4 weeks.

RT^2 lncRNA PCR Array Analysis

The RT^2 lncRNA PCR array system (https://www.qiagen.com/cn/shop/pcr/primer-sets/rt2-lncrna-pcr-arrays/?catno=LAHS-002Z#geneglobe) was applied to explore potential lncRNAs involved in the modulation of miR-200b expression in DTX-resistant LUAD cells. lncRNAs were considered differentially expressed in DTX-resistant LUAD cells for a fold change $\geq$ 2.0.

qRT-PCR Analysis

Total RNA was extracted by using TRIzol reagent (Invitrogen) according to the manufacturer's guidance. A PrimeScript RT reagent kit (Takara, Dalian, China) was used to carry out reverse transcription in accordance with the manufacturer's instructions. SYBR Prime Script RT-PCR kits (Takara) were used to perform qRT-PCR on the basis of the manufacturer's protocols. All mRNA expression levels were calculated by using the $2^{-\Delta\Delta Ct}$ method and were normalized to GAPDH or U6 expression. All assays were carried out independently three times. The PCR primers are listed in Table S1.

Luciferase Reporter Assay

pmirGLO; pmirGLO-WT for MALAT1, ZEB1, and E2F3; or pmirGLO-MUT for MALAT1, ZEB1, and E2F3 were cotransfected into SPC-A1/DTX or HEK293T cell lines, together with miR-200b mimics or miRNA normal controls, by using Lipofectamine-mediated gene transfer. Relative luciferase activity was normalized to Renilla luciferase activity 48 h after transfection. The data were relative to the

fold change of pair-matched control groups, which were defined as 1.0. All samples were assayed in triplicate.

In Vitro Chemosensitivity Assay

As previously described,[47] chemosensitivity was examined through MTT (Sigma, Natick, MA, USA) assays. Cells were incubated in 96-well plates and treated with DTX. After 48 h, each well was supplemented with MTT solution (5 mg/mL, 20 μL). After 4 h of incubation, the medium was removed, and 100 μL of DMSO was added to each well. The relative number of surviving cells was evaluated by measuring the cell lysate optical density at 560 nm (OD_{560}). All experiments were carried out independently three times.

Colony Formation Assay

Cells (500 cells/well) were put into 6-well plates and incubated in RPMI 1640 medium with 10% FBS at 37°C. 14 days later, the cells were fixed and dyed with 0.1% crystal violet.[48] The number of visible colonies was counted manually. All samples were assayed in triplicate.

EdU Incorporation Assays

EdU proliferation assays were performed by using Cell-Light EdU Apollo 567 *in vitro* imaging kits (Ribobio) according to the manufacturer's instructions. Cells (5×10^3 cells/wells) with the indicated treatment were seeded into 96-well plates. After 24 h, 100 μL of medium containing 50 μM EdU was added to each well, followed by incubation for 2 h at 37°C. Then, the cells were fixed with 4% paraformaldehyde and stained with a Hoechst and Apollo reaction cocktail. Images were captured using a fluorescence microscope (Nikon) and were merged using Adobe Photoshop 6.0 software. All experimental procedures were repeated at least three times.

Flow Cytometric Analysis

Flow cytometry analysis was carried out as previously described.[49,50] To analyze the cell apoptosis and cell-cycle distribution, cells were transfected with shCtrl or shMALAT1, treated with or without drugs for 24 h, and then collected. An annexin V/propidium iodide detection kit (KeyGen Biotech Co., Nanjing, China) was used for cell apoptosis assays. For cell-cycle analysis, cells were collected, fixed in 70% ethanol at 4°C for 16 h, and then stained with propidium iodide (PI). All experiments were carried out independently three times.

Immunofluorescence

Cells were seeded on glass coverslips in 6-well plates, fixed in 4% formaldehyde solution (Thermo Scientific), and permeabilized with 0.5% Triton X-100/PBS (Invitrogen). Cells were sealed with 5% BSA/PBS (Beyotime, Shanghai, China) for 1 h at room temperature and then were incubated with primary antibodies (E-cadherin and N-cadherin) at 4°C overnight, followed by incubation with fluorescent dye-conjugated secondary antibody (Invitrogen) for 1 h. Finally, the cells were stained with DAPI (Life Technologies, Gaithersburg, MD USA), and images were observed under a confocal microscope (TCS SP8 STED, Leica, Wetzlar, Germany).[51] All experiments were conducted independently three times.

RNA FISH

SPC-A1/DTX cells were seeded in glass chamber slides. 24 h after transfection, cells were fixed overnight in 4% paraformaldehyde (Thermo Scientific, Rockford, IL, USA), washed three times with PBS (5 min/wash), permeabilized with 0.5% Triton X-100/PBS permeation fluid at room temperature for 20 min, and again washed three times with PBS (5 min/wash). The slides were prehybridized for 1 h at 55°C with 100 mL of prehybridization buffer: 50% formamide (Sigma) + 5× saline sodium citrate (SSC) buffer + 5× Denhardt's solution + 0.5% Tween 20 (Thermo Scientific). Then, slides were washed three times (10 min/wash) in 0.1× SSC (Ambion) at 55°C, followed by three washes (2 min/wash) in 1× PBS (Gibco) at room temperature. Slides were blocked in 10% heat-inactivated goat serum + 0.5% blocking reagent in PBS-Tween for 1 h at room temperature. In addition, 150 mL of anti-FAM-POD (the polyclonal antibody reacts with free and bound fluorescein to reduce the non-specific binding), diluted 1:40 in blocking buffer, was added to each slide, and the slides were incubated for 1 h at room temperature. The slides were washed twice (2 min/wash) in 1× PBS, 50 mL of Cy3-TSA substrate was diluted at 1: 50, and the slides were incubated in the dark for 10 min at room temperature. Finally, the slides were mounted with DAPI (Life Technologies) nuclear stain.

Western Blot

All protein lysates were separated on 10% gels via SDS-PAGE and were electrophoretically transferred onto polyvinylidene difluoride membranes (Roche). Mouse anti-GAPDH monoclonal antibody was used to assess the protein loading. Membranes were blotted with 10% skim milk in Tris-buffered saline and Tween 20 (TBST) at room temperature for 2 h, followed by washing and probing with anti-E-cadherin (Abcam, Cat. no. ab133597, 1:1,000 dilution), and anti-GAPDH (Abcam, Cat. no. ab9485, 1:3,000 dilution) antibodies at 4°C overnight. Subsequently, the membranes were treated with secondary antibody conjugated to horseradish peroxidase for 2 h at room temperature. Proteins were detected with an enhanced chemiluminescence system and were exposed to X-ray film. All antibodies were purchased from Abcam (Cambridge, MA, USA). All samples were assayed in triplicate.

Subcellular Fractionation Assays

As previously described,[52] nuclear and cytosolic fraction separation was performed using a PARIS kit (Life Technologies, Carlsbad, CA, USA) according to the manufacturer's instructions.

RIP Assays

A Thermo Scientific RIP kit (Thermo, Waltham, MA, USA) was used to carry out RIP according to the manufacturer's instructions. Ago2 antibodies were purchased from Abcam. Normal mouse IgG (Abcam) was used as the negative control. Purified RNA was subjected to qRT-PCR analysis.

Chromatin Immunoprecipitation Assays

Cells were harvested for chromatin immunoprecipitation (ChIP) by using an EZ-ChIP kit based on the manufacturer's instructions

(Millipore, Burlington, MA, USA). ZEB1 and TFAP2C were obtained from Abcam. The ChIP primer sequences are listed in Table S1. Immunoprecipitated DNA was quantified using qPCR. Purified DNA was quantified using real-time qPCR.

Biotin-Promoter Pull-Down Assay and Mass Spectrometry

Double-stranded MALAT1 promoter was synthesized by PCR and labeled with biotin-14-dCTP under the manufacturer's protocol (Invitrogen, Grand Island, NY, USA). Biotin-labeled DNA was diluted in 10 mM Tris-HCl, 10 mM $MgCl_2$, 25 mM NaCl, and 10% glycerol and incubated with nuclear protein extract at 4°C for 12 h. In general, the biotinylated DNA was pull down by streptavidin-linked magnetic beads (Thermo Scientific) at room temperature for 2 h. The beads containing DNA and proteins were then washed four times with 1× binding and washing buffer. The proteins were precipitated and diluted in 100 μL of protein lysis buffer. One-shot mass spectrometry analyses were then performed to analyze the purified nuclear proteins. Triple TOF 6600 liquid chromatography-mass spectrometry (LC-MS) (AB Sciex, Shanghai, China) was used for mass spectrometry analyses.

Bioinformatics Analysis

Two binding sequences between MALAT1 and miR-200b were found from online bioinformatics analysis (http://starbase.sysu.edu.cn/). The possible targets of miR-200b were predicted using three bioinformatics prediction tools: TargetScan (http://www.targetscan.org/), RNA22 (http://www.rna-seqblog.com/rna22-version-2-0-mirna-mre-predictions/), and PicTar (http://pictar.mdc-berlin.de). The binding region of TFAP2C on the promoters of MALAT1 and ZEB1 and the binding region of ZEB1 on the promoter of MALAT1 were found from the University of California, Santa Cruz (UCSC) (http://genome.ucsc.edu/index.html).

Xenograft Transplantation and Immunohistochemical Assays

All nude mice (4–6 weeks old) were provided by the Comparative Medicine Department (Jinling Hospital, Nanjing, China). Before this procedure, the animal research received ethical approval in accordance with institutional guidelines. The ethical number is 2015NZGKJ-089. The subcutaneous xenograft model was established by directly subcutaneously injecting 2×10^6 SPC-A1/DTX cells stably transfected with shMALAT1 or shCtrl (n = 4 mice/group), which were previously suspended in 100 μL of PBS. When the average tumor size reached approximately 50 mm^3, DTX was given through intraperitoneal injections at a concentration of 1.0 mg/kg; one dose was provided every other day, with three total doses. After five weeks, all mice were killed, and necropsies were carried out. Primary tumors were excised, paraffin embedded and formalin fixed. H&E staining and Ki-67 immunostaining analysis were conducted, and using PCNA, the levels of E-cadherin and N-cadherin were evaluated according to the manufacturer's instructions.

For the tumor metastasis model, 1×10^6 SPC-A1/DTX-luciferase cells in 0.1 mL of PBS were injected into the tail vein of nude mice. Every 2–4 days, beginning 1 week after cell injection, DTX was injected intraperitoneally, given at a concentration of 1.0 mg/kg, with one dose every other day. Xenograft mouse images were obtained using a Xenogen IVIS 2000 Luminal Imager. After the mice were sacrificed, lungs were removed, and H&E staining was conducted. This study was carried out strictly in accordance with the Guide for the Care and Use of Laboratory Animals of the NIH. Our protocol was approved by the Committee on the Ethics of Animal Experiments of Jinling Hospital.

TUNEL Assay

In transplanted tumor tissues, apoptosis was measured by means of the TUNEL assay, which was carried out on the basis of guidelines for the TUNEL assay kit (KeyGen, Nanjing, China).

Statistical Analysis

SPSS v.21.0 software (IBM, Armonk, NY, USA) was applied for statistical analyses. Mean ± SD was used to present experimental results. Student's t test or one-way ANOVA was used to detect the differences among groups. p values < 0.05 were considered statistically significant.

SUPPLEMENTAL INFORMATION

Supplemental Information includes five figures and one table and can be found with this article online at https://doi.org/10.1016/j.omtn.2019.01.005.

AUTHOR CONTRIBUTIONS

J.C. and X.L. were responsible for the experimental design. The paper was drafted by J.C. and X.L. All authors gave valuable suggestions to this paper. The experiments were conducted by J.C. and X.L. All other authors helped to conduct the experiments. The experimental data were recorded and analyzed by all authors.

CONFLICTS OF INTEREST

The authors declare no competing interests.

ACKNOWLEDGMENTS

The authors thank all members involved in this study. This study was sponsored by the National Natural Science Foundation of China (grants 81572933, 81672928, and 81502611).

REFERENCES

1. Siegel, R.L., Miller, K.D., and Jemal, A. (2017). Cancer statistics, 2017. CA Cancer J. Clin. *67*, 7–30.
2. Siegel, R., Ma, J., Zou, Z., and Jemal, A. (2014). Cancer statistics, 2014. CA Cancer J. Clin. *64*, 9–29.
3. Jemal, A., Siegel, R., Xu, J., and Ward, E. (2010). Cancer statistics, 2010. CA Cancer J. Clin. *60*, 277–300.
4. Riaz, S.P., Lüchtenborg, M., Coupland, V.H., Spicer, J., Peake, M.D., and Møller, H. (2012). Trends in incidence of small cell lung cancer and all lung cancer. Lung Cancer *75*, 280–284.
5. Murray, N., and Turrisi, A.T., 3rd (2006). A review of first-line treatment for small-cell lung cancer. J Thorac Oncol *1*, 270–278.
6. Sarvi, S., Mackinnon, A.C., Avlonitis, N., Bradley, M., Rintoul, R.C., Rassl, D.M., Wang, W., Forbes, S.J., Gregory, C.D., and Sethi, T. (2014). CD133+ cancer stem-like

cells in small cell lung cancer are highly tumorigenic and chemoresistant but sensitive to a novel neuropeptide antagonist. Cancer Res. *74*, 1554–1565.

7. Joshi, M., Liu, X., and Belani, C.P. (2014). Taxanes, past, present, and future impact on non-small cell lung cancer. Anticancer Drugs *25*, 571–583.

8. Du, L., and Morgensztern, D. (2015). Chemotherapy for advanced-stage non-small cell lung cancer. Cancer J. *21*, 366–370.

9. Carninci, P., Kasukawa, T., Katayama, S., Gough, J., Frith, M.C., Maeda, N., Oyama, R., Ravasi, T., Lenhard, B., Wells, C., et al.; FANTOM Consortium; RIKEN Genome Exploration Research Group and Genome Science Group (Genome Network Project Core Group) (2005). The transcriptional landscape of the mammalian genome. Science *309*, 1559–1563.

10. Nagano, T., and Fraser, P. (2011). No-nonsense functions for long noncoding RNAs. Cell *145*, 178–181.

11. Zhou, C., Huang, C., Wang, J., Huang, H., Li, J., Xie, Q., Liu, Y., Zhu, J., Li, Y., Zhang, D., et al. (2017). lncRNA MEG3 downregulation mediated by DNMT3b contributes to nickel malignant transformation of human bronchial epithelial cells via modulating PHLPP1 transcription and HIF-1α translation. Oncogene *36*, 3878–3889.

12. Sun, Y., Wei, G., Luo, H., Wu, W., Skogerbø, G., Luo, J., and Chen, R. (2017). The long noncoding RNA SNHG1 promotes tumor growth through regulating transcription of both local and distal genes. Oncogene *36*, 6774–6783.

13. Chakravarty, D., Sboner, A., Nair, S.S., Giannopoulou, E., Li, R., Hennig, S., Mosquera, J.M., Pauwels, J., Park, K., Kossai, M., et al. (2014). The oestrogen receptor alpha-regulated lncRNA NEAT1 is a critical modulator of prostate cancer. Nat. Commun. *5*, 5383.

14. Ulitsky, I., and Bartel, D.P. (2013). lincRNAs: genomics, evolution, and mechanisms. Cell *154*, 26–46.

15. Batista, P.J., and Chang, H.Y. (2013). Long noncoding RNAs: cellular address codes in development and disease. Cell *152*, 1298–1307.

16. Mercer, T.R., Dinger, M.E., and Mattick, J.S. (2009). Long non-coding RNAs: insights into functions. Nat. Rev. Genet. *10*, 155–159.

17. Wilusz, J.E., Sunwoo, H., and Spector, D.L. (2009). Long noncoding RNAs: functional surprises from the RNA world. Genes Dev. *23*, 1494–1504.

18. Song, Y.X., Sun, J.X., Zhao, J.H., Yang, Y.C., Shi, J.X., Wu, Z.H., Chen, X.W., Gao, P., Miao, Z.F., and Wang, Z.N. (2017). Non-coding RNAs participate in the regulatory network of CLDN4 via ceRNA mediated miRNA evasion. Nat. Commun. *8*, 289.

19. Liang, H., Yu, T., Han, Y., Jiang, H., Wang, C., You, T., Zhao, X., Shan, H., Yang, R., Yang, L., et al. (2018). lncRNA PTAR promotes EMT and invasion-metastasis in serous ovarian cancer by competitively binding miR-101-3p to regulate ZEB1 expression. Mol. Cancer *17*, 119.

20. Cao, C., Zhang, T., Zhang, D., Xie, L., Zou, X., Lei, L., Wu, D., and Liu, L. (2017). The long non-coding RNA, SNHG6-003, functions as a competing endogenous RNA to promote the progression of hepatocellular carcinoma. Oncogene *36*, 1112–1122.

21. Zhang, S., Ma, H., Zhang, D., Xie, S., Wang, W., Li, Q., Lin, Z., and Wang, Y. (2018). lncRNA KCNQ1OT1 regulates proliferation and cisplatin resistance in tongue cancer via miR-211-5p mediated Ezrin/Fak/Src signaling. Cell Death Dis. *9*, 742.

22. Ren, J., Ding, L., Zhang, D., Shi, G., Xu, Q., Shen, S., Wang, Y., Wang, T., and Hou, Y. (2018). Carcinoma-associated fibroblasts promote the stemness and chemoresistance of colorectal cancer by transferring exosomal lncRNA H19. Theranostics *8*, 3932–3948.

23. Chen, X., Xie, R., Gu, P., Huang, M., Han, J., Dong, W., Xie, W., Wang, B., He, W., Zhong, G., et al. (2018). Long noncoding RNA LBCS inhibits self-renewal and chemoresistance of bladder cancer stem cells through epigenetic silencing of SOX2. Clin Cancer Res. Published online November 5, 2018. 10.1158/1078-0432.CCR-18-1656.

24. Feng, B., Wang, R., Song, H.Z., and Chen, L.B. (2012). MicroRNA-200b reverses chemoresistance of docetaxel-resistant human lung adenocarcinoma cells by targeting E2F3. Cancer *118*, 3365–3376.

25. Chen, D.Q., Pan, B.Z., Huang, J.Y., Zhang, K., Cui, S.Y., De, W., Wang, R., and Chen, L.B. (2014). HDAC 1/4-mediated silencing of microRNA-200b promotes chemoresistance in human lung adenocarcinoma cells. Oncotarget *5*, 3333–3349.

26. Chen, D.Q., Huang, J.Y., Feng, B., Pan, B.Z., De, W., Wang, R., and Chen, L.B. (2014). Histone deacetylase 1/Sp1/microRNA-200b signaling accounts for maintenance of cancer stem-like cells in human lung adenocarcinoma. PLoS ONE *9*, e109578.

27. Lv, J., Fan, H.X., Zhao, X.P., Lv, P., Fan, J.Y., Zhang, Y., Liu, M., and Tang, H. (2016). Long non-coding RNA Unigene56159 promotes epithelial-mesenchymal transition by acting as a ceRNA of miR-140-5p in hepatocellular carcinoma cells. Cancer Lett. *382*, 166–175.

28. Zhao, L., Han, T., Li, Y., Sun, J., Zhang, S., Liu, Y., Shan, B., Zheng, D., and Shi, J. (2017). The lncRNA SNHG5/miR-32 axis regulates gastric cancer cell proliferation and migration by targeting KLF4. FASEB J. *31*, 893–903.

29. Liu, H.T., Liu, S., Liu, L., Ma, R.R., and Gao, P. (2018). EGR1-mediated transcription of lncRNA-HNF1A-AS1 promotes cell-cycle progression in gastric cancer. Cancer Res. *78*, 5877–5890.

30. Prensner, J.R., and Chinnaiyan, A.M. (2011). The emergence of lncRNAs in cancer biology. Cancer Discov. *1*, 391–407.

31. Tsai, M.C., Manor, O., Wan, Y., Mosammaparast, N., Wang, J.K., Lan, F., Shi, Y., Segal, E., and Chang, H.Y. (2010). Long noncoding RNA as modular scaffold of histone modification complexes. Science *329*, 689–693.

32. Huang, M., Hou, J., Wang, Y., Xie, M., Wei, C., Nie, F., Wang, Z., and Sun, M. (2017). Long noncoding RNA LINC00673 is activated by SP1 and exerts oncogenic properties by interacting with LSD1 and EZH2 in gastric cancer. Mol. Ther. *25*, 1014–1026.

33. Ma, L., Sun, X., Kuai, W., Hu, J., Yuan, Y., Feng, W., and Lu, X. (2019). Retraction. DNA Cell Biol. *38*, 114.

34. Luo, Y., Chen, J.J., Lv, Q., Qin, J., Huang, Y.Z., Yu, M.H., and Zhong, M. (2019). Long non-coding RNA NEAT1 promotes colorectal cancer progression by competitively binding miR-34a with SIRT1 and enhancing the Wnt/β-catenin signaling pathway. Cancer Lett. *440*, 441, 11–22.

35. Shan, Y., Ma, J., Pan, Y., Hu, J., Liu, B., and Jia, L. (2018). lncRNA SNHG7 sponges miR-216b to promote proliferation and liver metastasis of colorectal cancer through upregulating GALNT1. Cell Death Dis. *9*, 722.

36. Wang, Y., Zeng, X., Wang, N., Zhao, W., Zhang, X., Teng, S., Zhang, Y., and Lu, Z. (2018). Long noncoding RNA DANCR, working as a competitive endogenous RNA, promotes ROCK1-mediated proliferation and metastasis via decoying of miR-335-5p and miR-1972 in osteosarcoma. Mol. Cancer *17*, 89.

37. Chen, Q.N., Chen, X., Chen, Z.Y., Nie, F.Q., Wei, C.C., Ma, H.W., Wan, L., Yan, S., Ren, S.N., and Wang, Z.X. (2017). Long intergenic non-coding RNA 00152 promotes lung adenocarcinoma proliferation via interacting with EZH2 and repressing IL24 expression. Mol. Cancer *16*, 17.

38. Yuan, S., Liu, Q., Hu, Z., Zhou, Z., Wang, G., Li, C., Xie, W., Meng, G., Xiang, Y., Wu, N., et al. (2018). Long non-coding RNA MUC5B-AS1 promotes metastasis through mutually regulating MUC5B expression in lung adenocarcinoma. Cell Death Dis. *9*, 450.

39. Zheng, Q., Cui, X., Zhang, D., Yang, Y., Yan, X., Liu, M., Niang, B., Aziz, F., Liu, S., Yan, Q., and Liu, J. (2017). miR-200b inhibits proliferation and metastasis of breast cancer by targeting fucosyltransferase IV and α1,3-fucosylated glycans. Oncogenesis *6*, e358.

40. Li, Z., Wu, X., Gu, L., Shen, Q., Luo, W., Deng, C., Zhou, Q., Chen, X., Li, Y., Lim, Z., et al. (2017). Long non-coding RNA ATB promotes malignancy of esophageal squamous cell carcinoma by regulating miR-200b/Kindlin-2 axis. Cell Death Dis. *8*, e2888.

41. Hu, L., Wu, Y., Tan, D., Meng, H., Wang, K., Bai, Y., and Yang, K. (2015). Up-regulation of long noncoding RNA MALAT1 contributes to proliferation and metastasis in esophageal squamous cell carcinoma. J. Exp. Clin. Cancer Res. *34*, 7.

42. Chang, J., Xu, W., Du, X., and Hou, J. (2018). MALAT1 silencing suppresses prostate cancer progression by upregulating miR-1 and downregulating KRAS. OncoTargets Ther. *11*, 3461–3473.

43. Pan, Y., Tong, S., Cui, R., Fan, J., Liu, C., Lin, Y., Tang, J., Xie, H., Lin, P., Zheng, T., et al. (2018). Long non-coding MALAT1 functions as a competing endogenous RNA to regulate vimentin expression by sponging miR-30a-5p in hepatocellular carcinoma. Cell Physiol. Biochem *50*, 108–120.

44. Li, S., Ma, F., Jiang, K., Shan, H., Shi, M., and Chen, B. (2018). Long non-coding RNA metastasis-associated lung adenocarcinoma transcript 1 promotes lung adenocarcinoma by directly interacting with specificity protein 1. Cancer Sci. *109*, 1346–1356.

45. Lu, G., Li, Y., Ma, Y., Lu, J., Chen, Y., Jiang, Q., Qin, Q., Zhao, L., Huang, Q., Luo, Z., et al. (2018). Long noncoding RNA LINC00511 contributes to breast cancer tumourigenesis and stemness by inducing the miR-185-3p/E2F1/Nanog axis. J. Exp. Clin. Cancer Res. *37*, 289.

46. Zeng, H., Wang, J., Chen, T., Zhang, K., Chen, J., Wang, L., Li, H., Tuluhong, D., Li, J., and Wang, S. (2019). Downregulation of long non-coding RNA Opa interacting protein 5-antisense RNA 1 inhibits breast cancer progression by targeting sex-determining region Y-box 2 by microRNA-129-5p upregulation. Cancer Sci. *110*, 289–302.

47. Tsang, W.P., and Kwok, T.T. (2009). The miR-18a* microRNA functions as a potential tumor suppressor by targeting on K-Ras. Carcinogenesis *30*, 953–959.

48. He, Z., Tang, F., Ermakova, S., Li, M., Zhao, Q., Cho, Y.Y., Ma, W.Y., Choi, H.S., Bode, A.M., Yang, C.S., and Dong, Z. (2008). Fyn is a novel target of (−)-epigallocatechin gallate in the inhibition of JB6 Cl41 cell transformation. Mol. Carcinog. *47*, 172–183.

49. Yang, F., Sarangarajan, R., Le Poole, I.C., Medrano, E.E., and Boissy, R.E. (2000). The cytotoxicity and apoptosis induced by 4-tertiary butylphenol in human melanocytes are independent of tyrosinase activity. J. Invest. Dermatol. *114*, 157–164.

50. Zhang, S.Z., Pan, F.Y., Xu, J.F., Yuan, J., Guo, S.Y., Dai, G., Xue, B., Shen, W.G., Wen, C.J., Zhao, D.H., and Li, C.J. (2005). Knockdown of c-Met by adenovirus-delivered small interfering RNA inhibits hepatocellular carcinoma growth *in vitro* and *in vivo*. Mol. Cancer Ther. *4*, 1577–1584.

51. Shi, D.M., Li, L.X., Bian, X.Y., Shi, X.J., Lu, L.L., Zhou, H.X., Pan, T.J., Zhou, J., Fan, J., and Wu, W.Z. (2018). miR-296-5p suppresses EMT of hepatocellular carcinoma via attenuating NRG1/ERBB2/ERBB3 signaling. J. Exp. Clin. Cancer Res. *37*, 294.

52. Wu, D.M., Wang, S., Wen, X., Han, X.R., Wang, Y.J., Shen, M., Fan, S.H., Zhang, Z.F., Shan, Q., Li, M.Q., et al. (2018). lncRNA SNHG15 acts as a ceRNA to regulate YAP1-Hippo signaling pathway by sponging miR-200a-3p in papillary thyroid carcinoma. Cell Death Dis. *9*, 947.

OMTN, Volume *14*

Supplemental Information

TFAP2C-Activated MALAT1 Modulates the Chemoresistance of Docetaxel-Resistant Lung Adenocarcinoma Cells

Jing Chen, Xiaobei Liu, Yichen Xu, Kai Zhang, Jiayuan Huang, Banzhou Pan, Dongqin Chen, Shiyun Cui, Haizhu Song, Rui Wang, Xiaoyuan Chu, Xiaoli Zhu, and Longbang Chen

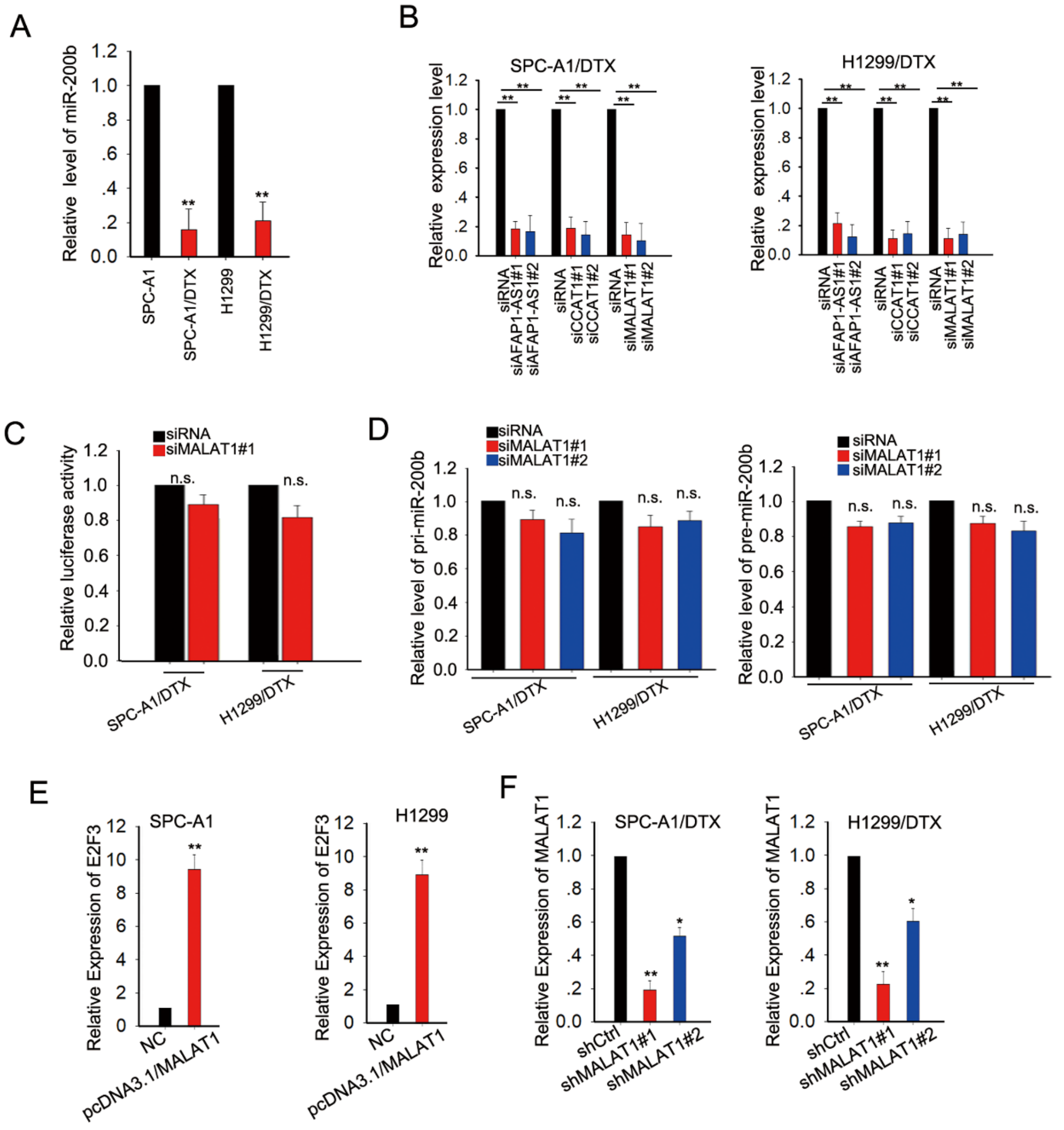

Supplementary Figure S1. The effects of

A. The miR-200b expression level in SPC-A1/DTX (H1299/DTX) and parental SPC-A1 (H1299) cells was determined by qRT-PCR. B. The knockdown efficiency of siRNAs specific to AFAP1-AS1, CCAT1 or MALAT1 was determined by qRT-PCR. C. The effect of MALAT1 knockdown on the miR-200b gene promoter activity was measured by luciferase reporter activity. D. The effect of MALAT1 knockdown on the expression levels pri-miR-200b and pre-miR-200b was measured by qRT-PCR. E. The transfection efficiency of pcDNA3.1/MALAT1 in parental cells and MALAT1-specific

shRNAs in DTX-resistant LUAD cells. **P < 0.01; n.s: no significance.

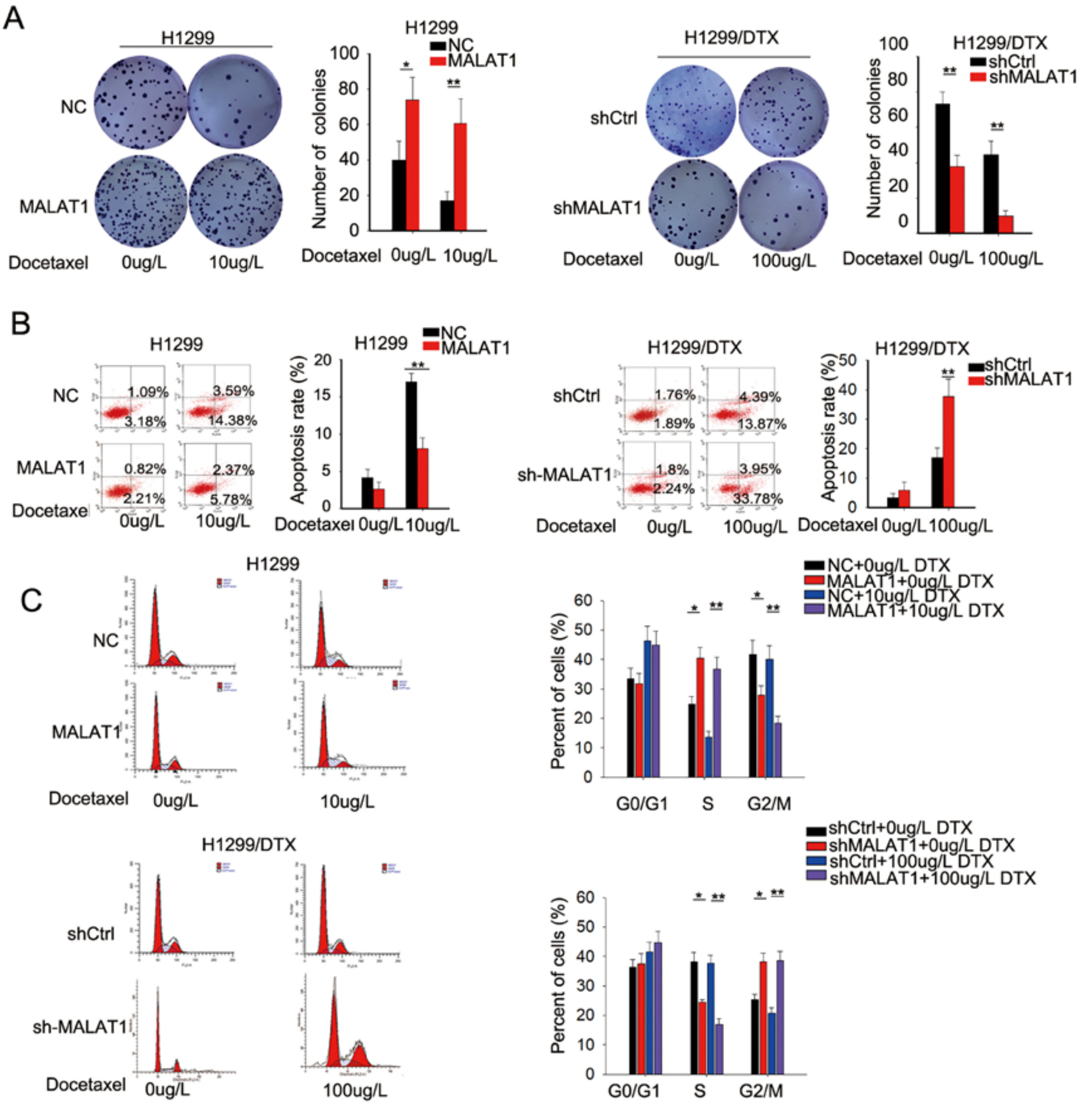

Supplementary Figure S2.

A. Representative colony formation results in H1299 cells transfected with MALAT1 expression vector and H1299/DTX cells transfected with MALAT1 shRNA. B-C. Flow cytometric analysis of apoptosis and cell cycle in H1299 cells transfected with pcDNA3.1 or pcDNA3.1/MALAT1 with or without treatment of DTX; or in H1299/DTX cells transfected with shRNA or shMALAT1 with or without treatment of DTX. *P < 0.05, **P < 0.01.

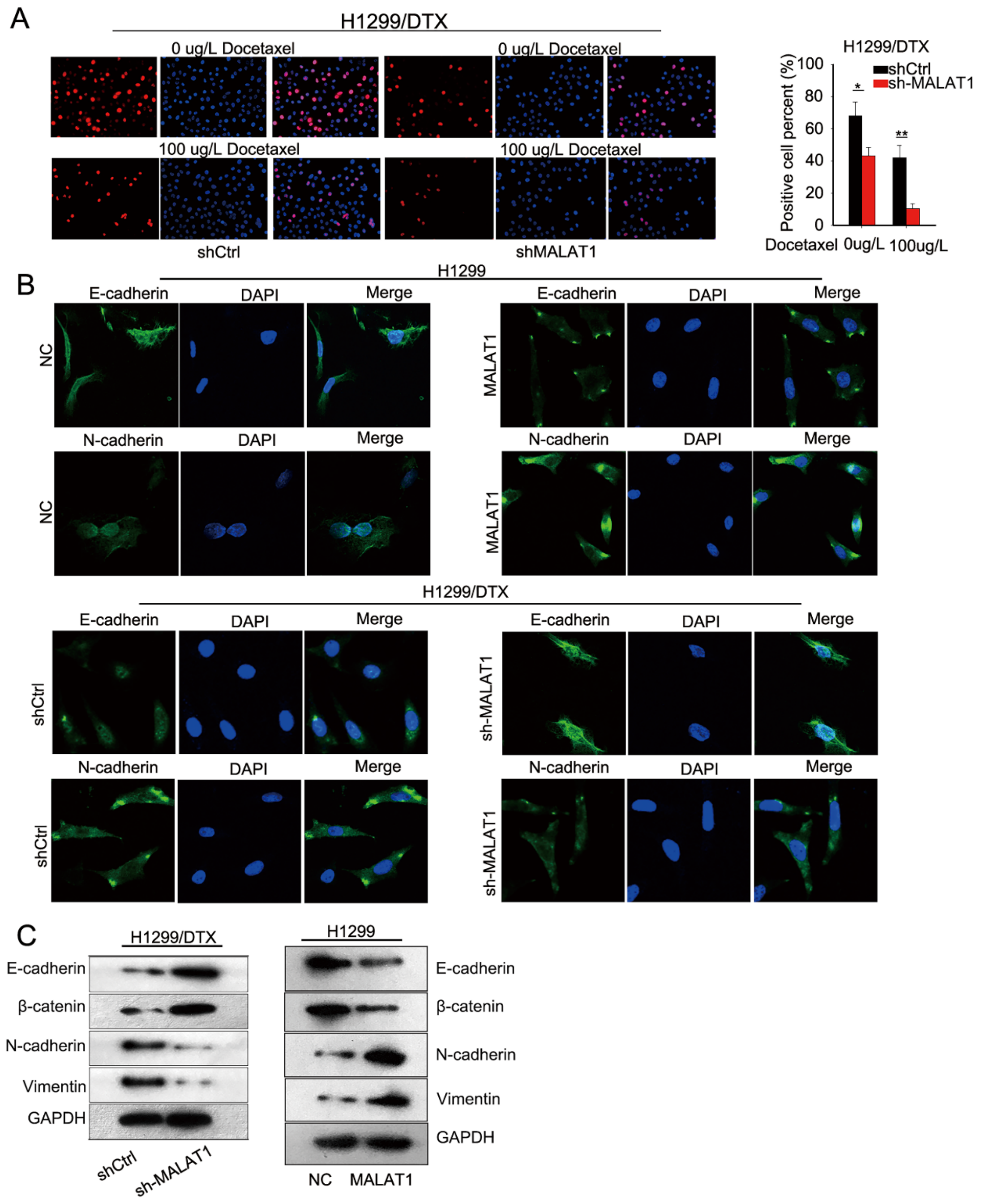

Supplementary Figure S3.

A. EdU proliferation assay in MALAT1-overexpressed H1299 cells or MALAT1-downregulated H1299/DTX cells. B. Immunofluorescence E-cadherin and N-cadherin staining was conducted in shRNA- or shMALAT1-transfected SPC-A1/DTX cells and pcDNA3.1- or pcDNA3.1/MALAT1-transfected SPC-A1 cells. C. E-cadherin and N-cadherin expression in shRNA- or shMALAT1-transfected H1299/DTX cells and

pcDNA3.1- or pcDNA3.1/MALAT1-transfected H1299 cells was determined by western blotting. *P < 0.05, **P < 0.01.

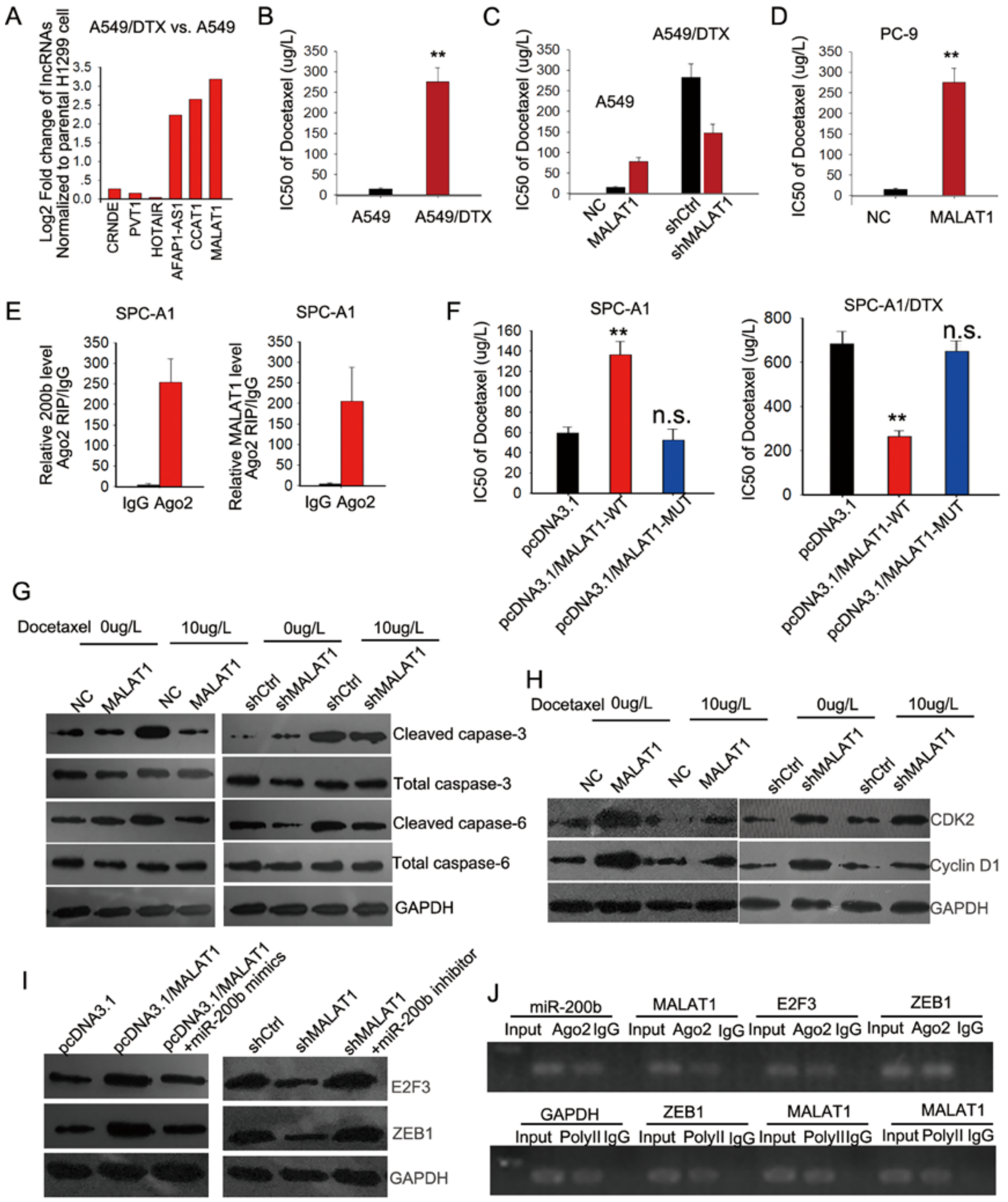

Supplementary Figure S4.

A. The fold change of six lncRNAs in A549/DTX and A549 cells. B. The IC_{50} of A549 and A549/DTX cells was tested with MTT assay. C. The IC_{50} was determined in A549

cell transfected with MALAT1 expression vector and NC or A549/DTX cell transfected with shMALAT1 and shCtrl. D. The IC_{50} value of PC-9 cell transfected with MALAT1 expression vector or NC. E. The interaction between MALAT1 and miR-200b in SPC-A1 cell was detected by RIP assay. F. The IC_{50} was examined in SPC-A1 cell transfected with pcDNA/MALAT1-WT, pcDNA/MALAT1-MUT or pcDNA-NC as well as in SPC-A1 cell transfected with shMALAT1-WT, shMALAT1-MUT or shCtrl. G-H. The expression levels of proteins related with cell cycle progress and apoptosis were tested by western blot analysis. I. The effects of MALAT1 and miR-200b on the protein levels of E2F3 and ZEB1 were determined by western blotting. J. Up: the gelling results for the qRT-PCR products of miRNA and mRNA; bottom: the DNA fragments for ChIP assay. **$P < 0.01$; n.s: no significance.

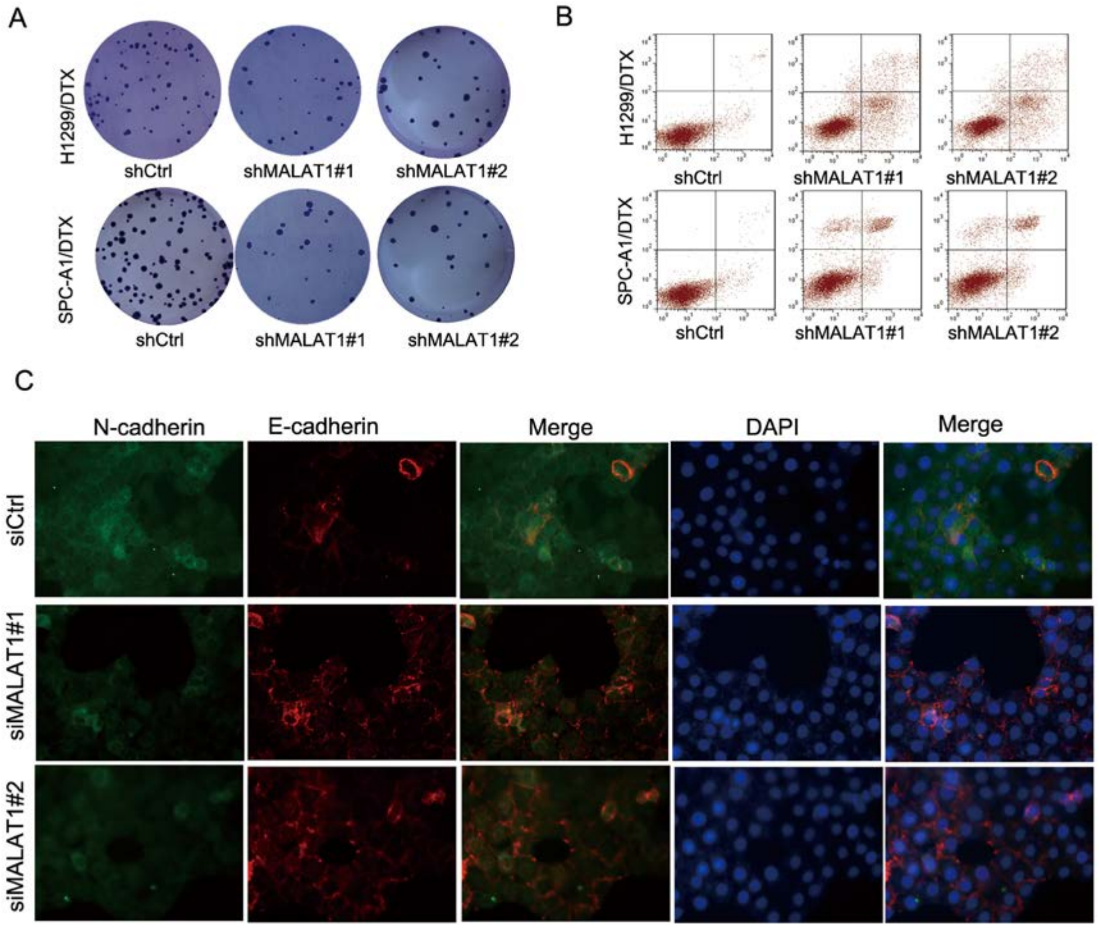

Supplementary Figure S5

A-C. Cell proliferation, apoptosis and EMT process were detected in SPC-A1/DTX and H1299/DTX cells transfected with shCtrl, shMALAT1#1 and shMALAT1#2.
